# Musculocontractural Ehlers–Danlos syndrome and neurocristopathies: dermatan sulfate is required for *Xenopus* neural crest cells to migrate and adhere to fibronectin

**DOI:** 10.1242/dmm.024661

**Published:** 2016-06-01

**Authors:** Nadège Gouignard, Marco Maccarana, Ina Strate, Kristoffer von Stedingk, Anders Malmström, Edgar M. Pera

**Affiliations:** 1 Department of Laboratory Medicine, Lund Stem Cell Center, Lund University, Lund 221 84, Sweden; 2 Department of Experimental Medical Science, Lund University, Lund 221 84, Sweden; 3 Department of Pediatrics, Lund University, Lund 223 63, Sweden

**Keywords:** Cell migration, Neural crest, Dermatan sulfate, Musculocontractural Ehlers–Danlos syndrome, Cancer, *Xenopus*

## Abstract

Of all live births with congenital anomalies, approximately one-third exhibit deformities of the head and face. Most craniofacial disorders are associated with defects in a migratory stem and progenitor cell population, which is designated the neural crest (NC). Musculocontractural Ehlers–Danlos syndrome (MCEDS) is a heritable connective tissue disorder with distinct craniofacial features; this syndrome comprises multiple congenital malformations that are caused by dysfunction of dermatan sulfate (DS) biosynthetic enzymes, including DS epimerase-1 (DS-epi1; also known as DSE). Studies in mice have extended our understanding of DS-epi1 in connective tissue maintenance; however, its role in fetal development is not understood. We demonstrate that DS-epi1 is important for the generation of isolated iduronic acid residues in chondroitin sulfate (CS)/DS proteoglycans in early *Xenopus* embryos. The knockdown of DS-epi1 does not affect the formation of early NC progenitors; however, it impairs the correct activation of transcription factors involved in the epithelial–mesenchymal transition (EMT) and reduces the extent of NC cell migration, which leads to a decrease in NC-derived craniofacial skeleton, melanocytes and dorsal fin structures. Transplantation experiments demonstrate a tissue-autonomous role for DS-epi1 in cranial NC cell migration *in vivo*. Cranial NC explant and single-cell cultures indicate a requirement of DS-epi1 in cell adhesion, spreading and extension of polarized cell processes on fibronectin. Thus, our work indicates a functional link between DS and NC cell migration. We conclude that NC defects in the EMT and cell migration might account for the craniofacial anomalies and other congenital malformations in MCEDS, which might facilitate the diagnosis and development of therapies for this distressing condition. Moreover, the presented correlations between human DS-epi1 expression and gene sets of mesenchymal character, invasion and metastasis in neuroblastoma and malignant melanoma suggest an association between DS and NC-derived cancers.

## Abstract (precis)

**Editors' choice:** In the *Xenopus* neural crest, dermatan sulfate is essential for cell migration *in vivo* and cell adhesion to fibronectin, which might have implications for musculocontractural Ehlers–Danlos syndrome and cancer.

## INTRODUCTION

The musculocontractural type of Ehlers–Danlos syndrome (MCEDS) is characterized by distinct craniofacial features, multisystem congenital malformations and progressive fragility of connective tissues (Zhang et al., 2010; Kosho, 2016). This rare intractable disorder is caused by recessive loss-of-function mutations in genes that encode dermatan sulfate (DS) biosynthetic enzymes, including dermatan 4-O-sulfotransferase 1 (CHST14) and DS epimerase-1 (DS-epi1). Glycosaminoglycans (GAGs), such as DS, chondroitin sulfate (CS) and heparan sulfate (HS), are side chains of repeating disaccharides, which are covalently attached to distinct core proteins in the Golgi complex to form cell surface and extracellular matrix proteoglycans (PGs) (Iozzo and Schaefer, 2015). DS is formed from CS by the partial conversion of glucuronic acid (GlcA) into iduronic acid (IdoA) (Trowbridge and Gallo, 2002; Thelin et al., 2013). The content of IdoA is variable and ranges from one IdoA residue per chain to nearly 100% IdoA; thus, the name CS/DS is assigned to describe the hybrid nature of the chain. DS-epi1 and DS-epi2, which are encoded by *Dse* and *Dse-like* (*Dsel*), respectively, mediate the epimerization of a carboxyl group at C5 to form IdoA in CS/DS (Maccarana et al., 2006; Pacheco et al., 2009b). *Dse*-knockout mice have connective tissue fragility attributed to a CS/DS alteration of decorin, which results in impaired assembly of collagen fibrils (Maccarana et al., 2009; Gustafsson et al., 2014), and a series of malformations whose cause is not understood. In contrast, the loss of *Dsel* results in normal offspring (Bartolini et al., 2012). Double-knockout mice die around birth and completely lack IdoA in their CS/DS chains, which indicates that DS-epi1 and DS-epi2 are the only epimerases in DS biosynthesis (Stachtea et al., 2015). DS-epi1, which was originally referred to as squamous cell carcinoma antigen recognized by cytotoxic T lymphocytes 2 (SART2) (Nakao et al., 2000; Maccarana et al., 2006), is overexpressed in all tumors and functionally linked to the tumorigenic properties of esophagus squamous cell carcinoma that involve cell migration (Thelin et al., 2012). Nevertheless, it is not clear how a modified CS/DS chain leads to developmental abnormalities and malignancy.

To investigate the function of IdoA in CS/DS chains at the cellular level, we searched for an appropriate model system. Craniofacial anomalies constitute a high proportion of congenital malformations and are mainly caused by neural crest (NC) development defects (Gorlin et al., 1990). Interestingly, the craniofacial features in MCEDS (Müller et al., 2013; Syx et al., 2015; Kosho, 2016) are reminiscent of NC-associated disorders, such as Treacher Collins syndrome, Nager syndrome and Miller syndrome (Trainor and Andrews, 2013), which indicates that the NC might be a suitable model system for MCEDS. The NC comprises a population of multipotent and highly migratory cells that form at the border between the neural and epidermal ectoderm in the vertebrate embryo (Mayor and Theveneau, 2013; Simões-Costa and Bronner, 2015). Research predominately in *Xenopus* and the chick embryo has demonstrated that signaling molecules secreted from the surrounding ectoderm and the underlying mesoderm, including bone morphogenetic proteins, Wnts and fibroblast growth factors (FGFs), orchestrate a combinatorial expression of transcription factors that drive NC specification and morphogenesis. NC cells undergo an epithelial–mesenchymal transition (EMT), migrate along restricted pathways through the embryo and contribute to nearly every organ system in the body, including the craniofacial skeleton, melanocytes, endocrine cells and the peripheral nervous system. Developmental disturbances in the NC, which are collectively referred to as neurocristopathies, encompass defects in NC specification, migration and differentiation, and include tumors of NC lineages, such as neuroblastoma and melanoma (Zhang et al., 2014). EMT and cell migration are hallmarks of both NC development and cancer metastasis (Powell et al., 2013).

As a result of their large embryo size and external development, *Xenopus* is a favorable experimental system. Here, we demonstrate that DS-epi1 accounts for most DS biosynthesis in the early *Xenopus* embryo. In loss-of-function assays, DS-epi1 is required for the correct regulation of neural-plate-border- and NC-specific transcription factors. Moreover, DS-epi1 has an intrinsic role in NC cell migration and is indispensable for the cell adhesion, spreading and formation of polarized cell structures on fibronectin. Human *DSE* expression correlates with genetic markers of EMT, invasion and metastasis in both neuroblastoma and melanoma, which suggests a potential role of DS-epi1 in NC-derived cancers. A model is proposed, in which CS/DS PGs mediate the adherence of NC cells to fibronectin during cell migration.

## RESULTS

### *Dse* and *Dsel* are expressed in the early *Xenopus* embryo

**Fig. 1.**
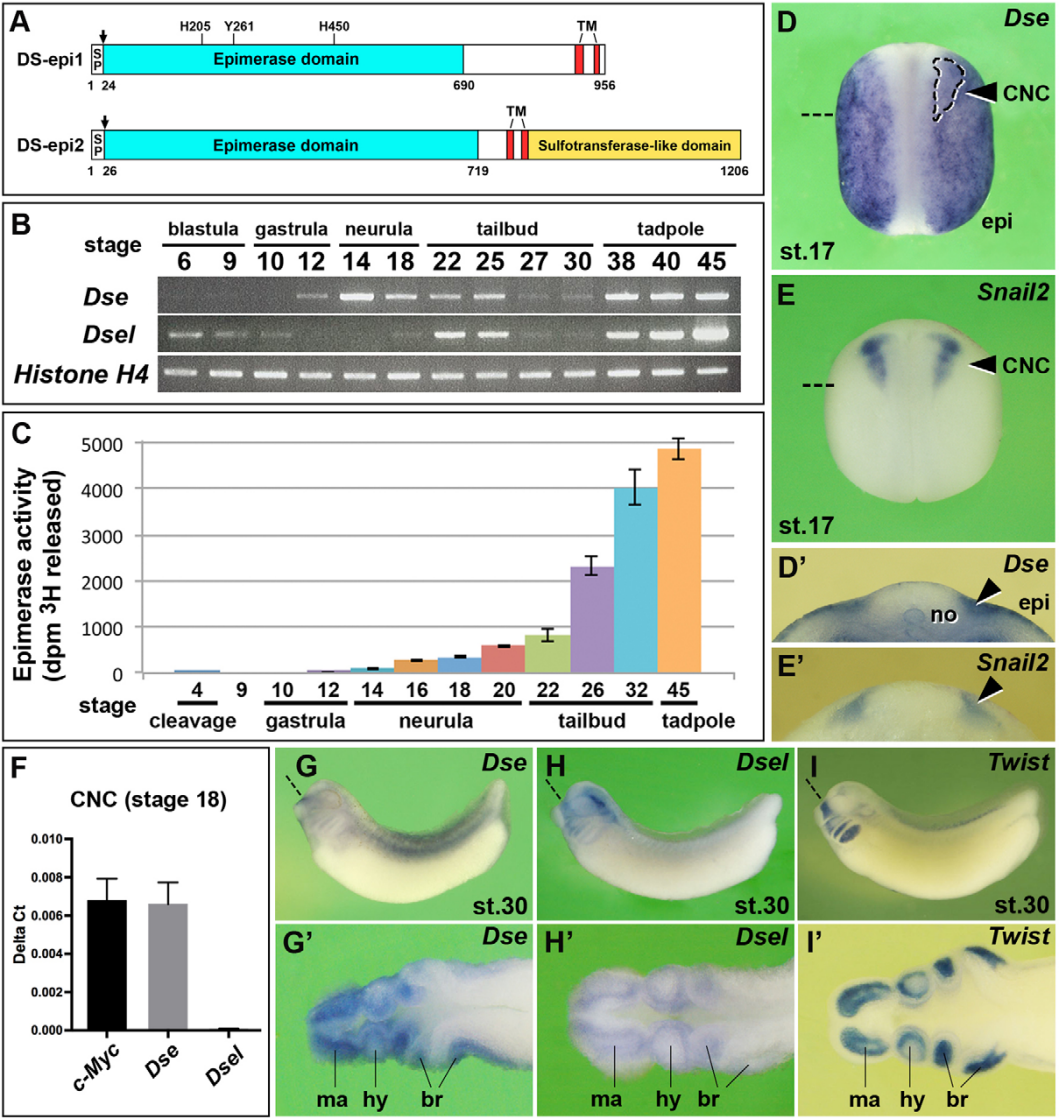
**Expression and activity of the two dermatan sulfate epimerases in *Xenopus* embryos.** (A) Protein structures. *Xenopus* DS-epi1 and DS-epi2 contain cleavable signal peptides (SP, arrows), an epimerase domain and two transmembrane (TM) domains. In DS-epi1, the catalytic residues His205, Tyr261 and His450 are indicated, which are also conserved in DS-epi2. DS-epi2 contains an additional sulfotransferase-like domain. (B) RT-PCR analysis of *Dse* and *Dsel*. *Histone H4* is used as the loading control. A minimum of two experiments (*n*≥2) was performed with three independent biological samples. (C) DS epimerase activity in early *Xenopus* embryos. Results are mean±s.d. from triplicates (two independent experiments). (D-E′) Whole-mount *in situ* hybridization of neurula embryos in the dorsal view (D,E) and in transversal section (D′,E′). The arrowheads label the pre-migratory CNC cells. The region enclosed by the dashed line demarcates the *Snail2*^+^ CNC embedded in the *Dse* expression domain. (F) qPCR analysis in CNC explants at stage 18. *c-Myc* was used as a CNC cell marker. Note that *Dse* but not *Dsel* mRNA is detected. Results are mean±s.d. from triplicates (*n*=4 biological replicates). (G-I′) Tailbud embryos in the lateral view (G-I) and horizontal section (G′-I′). Note that *Dse* and *Dsel* overlap with *Twist* expression in migrating CNC cells. Section planes are indicated with dashed straight lines. br, branchial arch segments; epi, epidermis; hy, hyoid segment; ma, mandibular segment; no, notochord.

We have previously demonstrated that intra-blastocoelic injection of purified DS, but not CS, stimulates posterior development, mesoderm formation and neuronal differentiation in an FGF-dependent manner (Hou et al., 2007). Enzymatic degradation of endogenous DS yielded opposite results, which suggests an important role of this GAG in early *Xenopus* embryos. To better understand the biosynthesis and function of DS, we focused on DS-epi1 and DS-epi2. Using a BLAST search in Xenbase for each gene, we identified two *Xenopus laevis* homeologs that differ in the derived amino acid sequences by 6% between DS-epi1.S and DS-epi1.L (Fig. S1), and 4% between DS-epi2.S and DS-epi2.L (Fig. S2), and also have well-conserved orthologs in other vertebrates. DS-epi1 and DS-epi2 share a cleavable signal peptide followed by an epimerase domain with 49% identity and two transmembrane domains (Fig. 1A). At the C-terminus, DS-epi2 contains a conserved sulfotransferase-like domain.

RT-PCR demonstrated that *Dse* expression was initiated in late gastrula embryos (Fig. 1B). *Dsel* was maternally expressed, and abundant zygotic transcripts were identified from the early tailbud stage onwards. DS epimerase activity represents the sum of DS-epi1 and DS-epi2 contributions (Maccarana et al., 2006). In *Xenopus*, there was a minute maternal level of epimerase activity and an onset of zygotic activity at the late gastrula stage, with a subsequent constant increase until at least stage 45 (Fig. 1C). Whole-mount *in situ* hybridization of stage 17 embryos indicated *Dse* expression in the epidermis, notochord and pre-migratory NC that overlapped with *Snail2* expression in the cranial neural crest (CNC) (Fig. 1D-E′). Quantitative real-time PCR (qPCR) analysis confirmed *Dse* but not *Dsel* mRNA in isolated *c-Myc*^+^ CNC explants from stage 18 embryos (Fig. 1F). At stage 30, *Dse* and *Dsel* expression coincided with migrating *Twist*^+^ CNC cells in the mandibular, hyoid and branchial arches (Fig. 1G-I′).

### DS-epi1 is the main contributor to early DS biosynthesis and is required for NC-derived structures

**Fig. 2.**
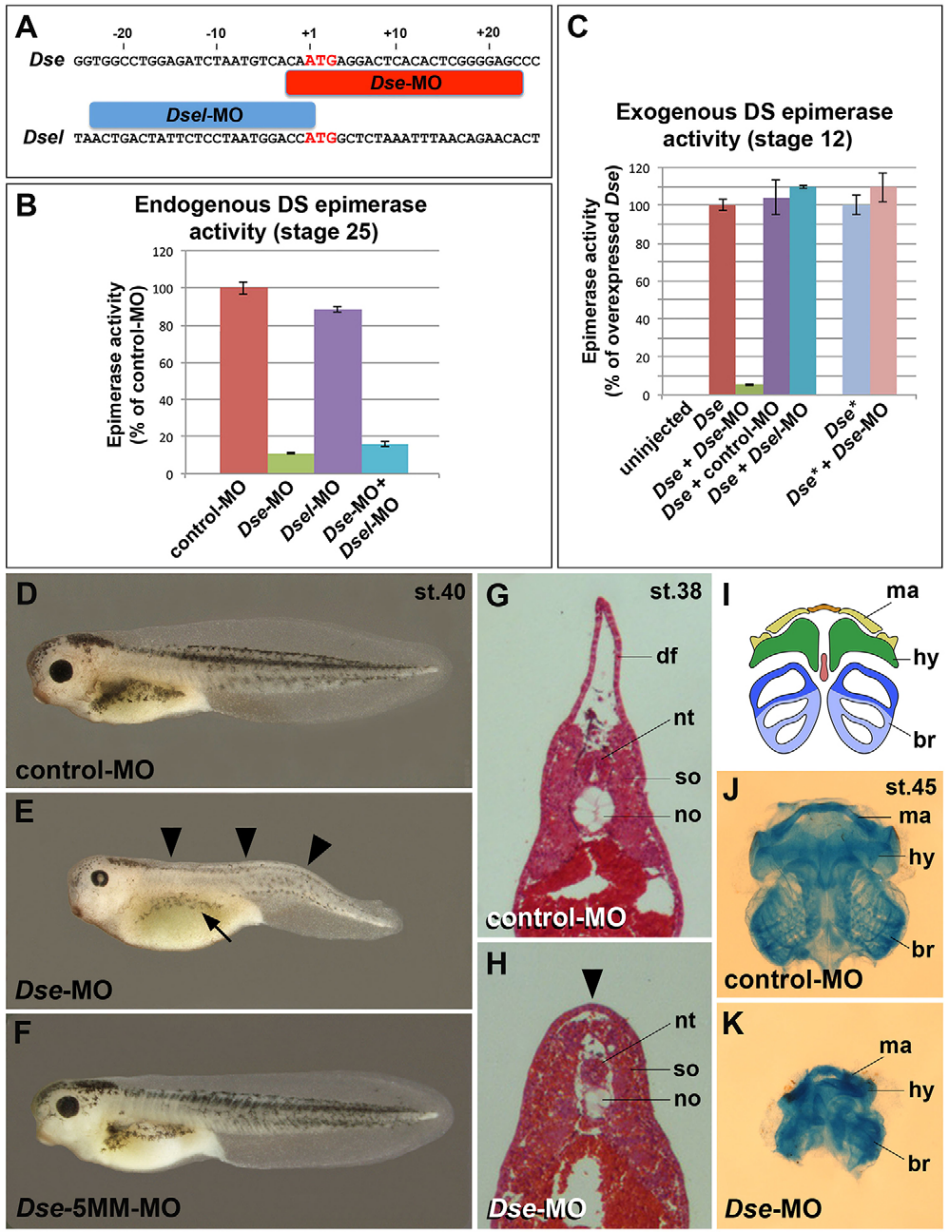
**Knockdown of DS-epi1 reduces DS epimerase activity and NC-derived structures.** (A) Morpholino oligonucleotides target the translation initiation sites of *Dse* and *Dsel*. (B) Endogenous DS epimerase activity is substantially decreased by *Dse*-MO but only a little by *Dsel*-MO in stage 25 embryos. (C) Epimerase activity induced by the injection of 1 ng *Dse* mRNA is blocked by *Dse*-MO, but not by control-MO and *Dsel*-MO. The activity of 1 ng non-targeted Dse* mRNA is not affected by *Dse*-MO. Results in B and C are mean±s.d. (*n*=3). (D) Tadpole at stage 40 injected with control-MO. (E,F) Microinjection of *Dse*-MO, but not *Dse*-5MM-MO, induces small eyes, a lack of dorsal fin structures (arrowheads) and reduced melanocyte formation (arrow). (G,H) Transversal trunk sections of stage 38 embryos following hematoxylin and eosin staining. Note the lack of a dorsal fin (arrowhead), dorsally approaching somites and hypoplastic notochord in the *Dse*-morphant embryo. (I-K) Ventral view of head skeletons at stage 45 in a schematic overview (I) and following Alcian Blue staining (J,K). Injection of *Dse*-MO, but not control-MO, causes a reduction of NC-derived cartilage structures. br, branchial segment; hy, hyoid segment; df, dorsal fin; ma, mandibular segment; no, notochord; nt, neural tube; so, somite. The proportion of examined tadpoles or explants with the indicated phenotype was as follows: D, 70/70; E, 71/114 (microcephaly), 92/114 (reduced dorsal fin), 70/114 (less melanocytes); F, 63/63; G, 4/4; H, 4/4; J, 25/25; and K, 20/20.

To investigate the relative contribution of DS-epi1 and DS-epi2 to epimerase activity in the embryo, we used morpholino oligonucleotides (MOs) against *Dse* and *Dsel* (Fig. 2A). Following microinjection into the animal pole of embryos (hereafter called animal injection), *Dse*- and *Dsel*-MOs blocked 89% and 12% of the endogenous DS epimerase activity, respectively, at stage 25 (Fig. 2B). The *Dse*-MO substantially suppressed the epimerase activity induced by injected *Dse* mRNA at stage 12, whereas a standard control MO and a *Dsel*-MO had no effect (Fig. 2C). In contrast, the *Dse*-MO did not block the epimerase activity of the injected Dse* mRNA, which contains seven point mutations in the MO target sequence; this finding demonstrates the specificity of the *Dse*-MO knockdown. The data suggest that DS-epi1 is the main contributor to DS biosynthesis in the early *Xenopus* embryo.

Animal injection of *Dse*-MO caused a decreased number of melanocytes, a reduction of head and eye structures, a lack of dorsal fin tissue and a kinked shortened tail in stage 40 embryos (Fig. 2E). Both the control-MO and *Dse*-5MM-MO, which contains five base mismatches with the *Dse* target mRNA, had no effect (Fig. 2D,F). Histological analysis at stage 38 indicated that *Dse*-morphant tadpoles had a hypoplastic notochord and somite structures that were dorsally abutting above the neural tube (Fig. 2G,H). Moreover, *Dse* knockdown led to a reduction in the mandibular, hyoid and branchial cartilage in the head at stage 45 (Fig. 2I-K). Interestingly, defects in melanocyte, craniofacial skeleton and dorsal fin formation are characteristic of deficient NC formation (Tucker, 1986; Sadaghiani and Thiébaud, 1987; Tucker and Slack, 2004), which suggests that DS-epi1 is required for NC development. Animal injection of *Dse* mRNA had no apparent effect on embryonic development at stage 40 and did not affect the formation or migration of *Twist*^+^ CNC cells at stages 16 and 26, respectively (Fig. S3).

### Isolated IdoA residues are present in CS/DS PGs in early embryos

**Fig. 3.**
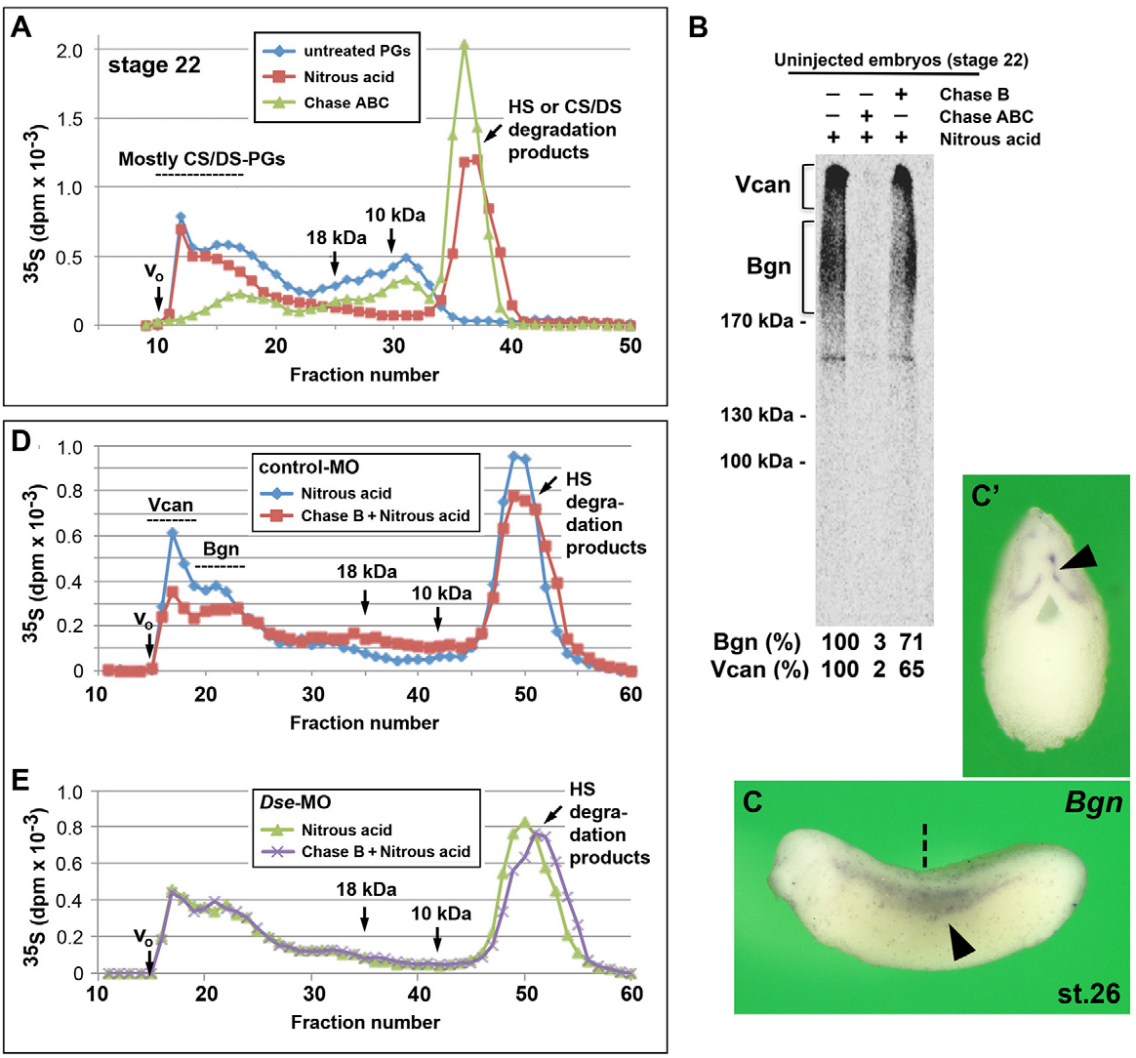
**Presence of IdoA in CS/DS PGs of early embryos.** (A) At stage 22, IdoA is present in high molecular mass CS/DS PGs, as demonstrated by the size-fractionation of [^35^S]-containing PGs. The HS and CS/DS degradation products are produced by nitrous acid and Chase ABC treatment, respectively. CS/DS PGs represent 72% of the high molecular mass PGs (fractions 12-17). (B) SDS-PAGE analysis of [^35^S]-labeled CS/DS PGs. The same samples analyzed by gel filtration in A were separated using a 4-10% gradient SDS-PAGE following nitrous acid or, alternatively, Chase ABC or Chase B treatments. The fluography indicates CS/DS PGs (brackets) with an apparent molecular mass of 200-300 kDa (Bgn) and ∼1000 kDa (Vcan). The percentages of radioactivity in the framed areas are indicated below each lane. (C,C′) Whole-mount *in situ* hybridization of *Bgn* at stage 26. Embryo is shown in the lateral view (C) and transversally sectioned (C′). Arrowheads indicate migrating trunk neural crest cells. The section planes are indicated by the dashed straight line. (D,E) Chase B treatment degrades CS/DS chains in high molecular mass PGs in control-MO-injected embryos (D) but not *Dse*-MO-injected embryos (E). Bgn, biglycan; Chase, chondroitinase; Vcan, versican.

To investigate the PG expression and GAG composition in *Xenopus* embryos, we microinjected [^35^S]sulfate into the blastocoel at stage 9 and analyzed the purified PGs at stage 22 after chemical and lyase treatment to degrade distinct GAG chains (Fig. 3). Nitrous acid at low pH was used to degrade HS (Shively and Conrad, 1976), and chondroitinases that are specific for DS (denoted Chase B) or both CS and DS (denoted Chase ABC) were used to degrade the DS or CS/DS chains, respectively. The size separation of the split products indicated that 56% of the total radioactivity corresponded to CS/DS PGs (Fig. 3A). SDS-PAGE analysis demonstrated two broad bands that were resistant to nitrous acid treatment and compatible with being biglycan (Bgn, 200-300 kDa, Moreno et al., 2005) and versican (Vcan, ∼1000 kDa) (Fig. 3B). Both bands disappeared following Chase ABC digestion and decreased following Chase B digestion to 71% (Bgn) and 65% (Vcan). We have previously demonstrated that CS/DS chains are present in *Xenopus* overexpressing *Bgn* (Hou et al., 2007). In post-neurula embryos, both *Bgn* (Fig. 3C,C′) and *Vcan* (Casini et al., 2008) are expressed in the trunk NC. Chase B treatment demonstrated IdoA in the high molecular mass fractions of control-MO- but not *Dse*-MO-injected embryos (Fig. 3D,E), which underscores the crucial role of DS-epi1 in DS biosynthesis. The Chase-B-degraded products primarily moved to fractions of 10-20 kDa, which indicates that IdoA is predominately a rare and isolated structure that is surrounded by long stretches of GlcA. We conclude that embryos at the early tailbud stage synthesize high molecular mass CS/DS PGs, such as Bgn and Vcan, with only few IdoA residues per chain.

### Biosynthesis of DS is not regulated by feedback

We subsequently investigated whether the presence of IdoA in CS/DS PGs affects the gene expression of DS epimerases. qPCR analysis indicated no changes in the *Dse* and *Dsel* mRNA levels in stage 18 CNC explants of the *Dse*-MO- compared with the *Dse*-5MM-MO-injected control embryos (Fig. S4). This result argues against feedback regulation of IdoA in CS/DS biosynthesis at the transcriptional level.

### DS-epi1 regulates the expression of neural plate border and cranial neural crest markers

**Fig. 4.**
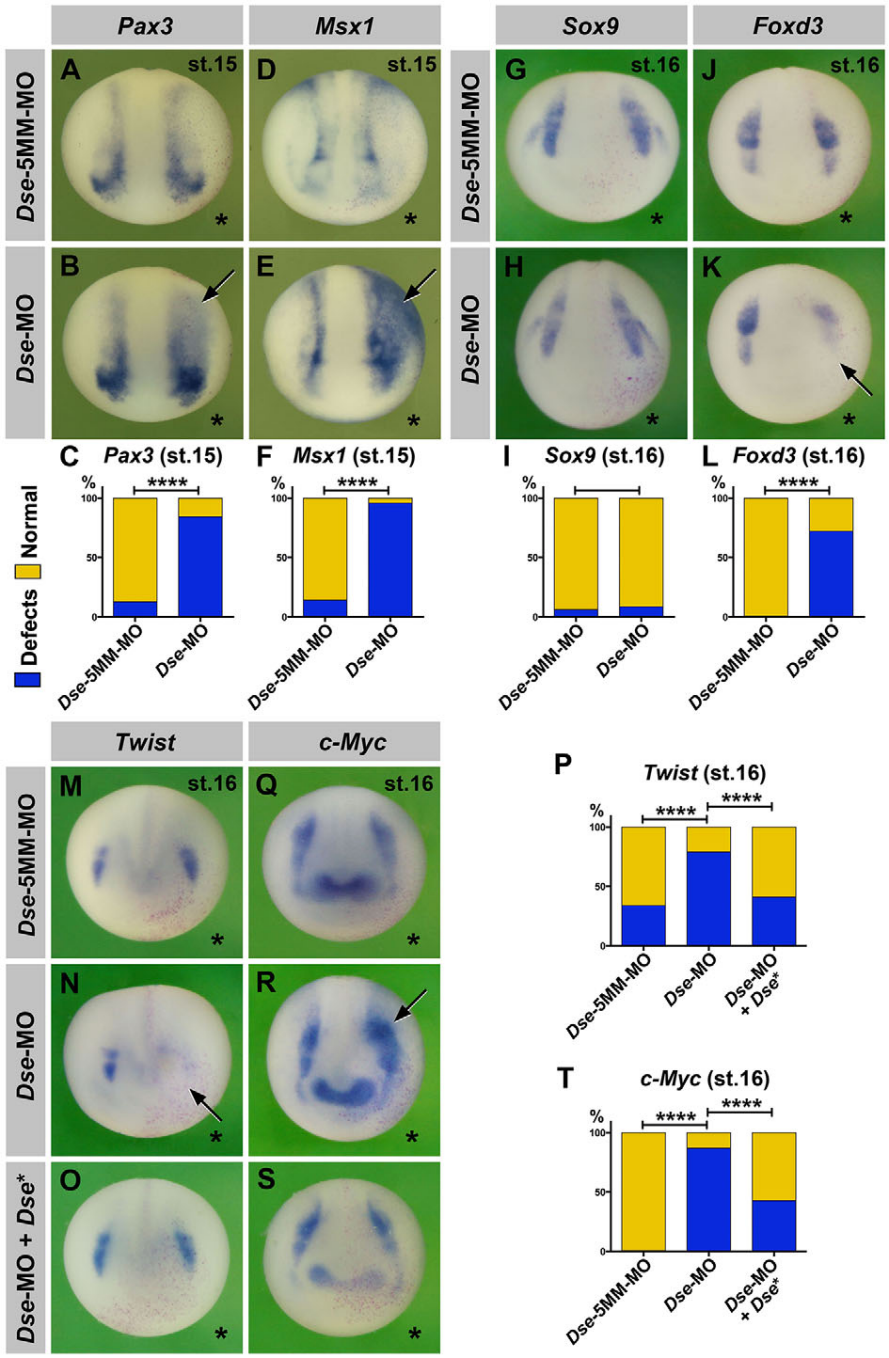
**DS-epi1 regulates gene markers of the neural plate border and CNC.** Whole-mount *in situ* hybridization of early neurula embryos in an anterior view. The injected side is marked with a star. (A-F) A single injection of *Dse*-MO into embryos causes expansion of *Pax3* and *Msx1* expression at the neural plate border (arrows). The *Dse*-5MM-MO has no effect. A quantification of the percentage of embryos with defects is shown in C and F. (G-T) *Dse*-MO has no significant effect on *Sox9*; however, it triggers a reduction in *Foxd3* and *Twist* expression, as well as an expansion of *c-Myc* expression (arrows). Normal *Twist* and *c-Myc* expression is restored by the co-injection of *Dse*-MO and 250 pg Dse* mRNA. *nlacZ* mRNA was injected as a lineage tracer (red nuclei). A quantification of the percentage of embryos with defects is shown in I, L, P and T. The proportion of examined embryos with the indicated phenotype was as follows: A, 37/42; B, 32/38; D, 26/30; E, 30/31; G, 35/37; H, 58/63; J, 30/30; K, 26/36; M, 13/20; N, 48/61; O, 16/27; Q, 90/90; R, 77/89; and S, 15/26. ****P<0.0001 (Fisher's exact test with two-tailed *P*-value calculation).

Animal injection of *Dse*-MO did not affect the extent of the *Sox2*^+^ neural plate or *Cytokeratin*^+^ epidermis (Fig. S5A,B); however, it expanded the expression domains of the neural plate border markers *Pax3* and *Msx3* into the ventral ectoderm at stage 15 (Fig. 4B,C,E,F). DS-epi1 knockdown had no substantial effect on the *Sox9* expression in the pre-migratory CNC (Fig. 4H,I); however, it reduced the levels of both the *Foxd3* and *Twist* markers (Fig. 4K,L,N,P) and concomitantly expanded the *c-Myc* expression in these cells (Fig. 4R,T). The *Dse*-5MM-MO had no effect (Fig. 4A,D,G,J,M,Q), and the co-injection of Dse* mRNA and *Dse*-MO reverted the reduction of *Twist* and the expansion of the *c-Myc* expression (Fig. 4O,S,P,T), which supports the specificity of the phenotypes. Thus, DS-epi1 is not involved in the early dorsoventral ectoderm patterning; however, it controls the expression of neural-plate-border- and distinct CNC-specific transcription factors in neurula embryos.

### DS-epi1 is important for cranial neural crest cell migration

**Fig. 5.**
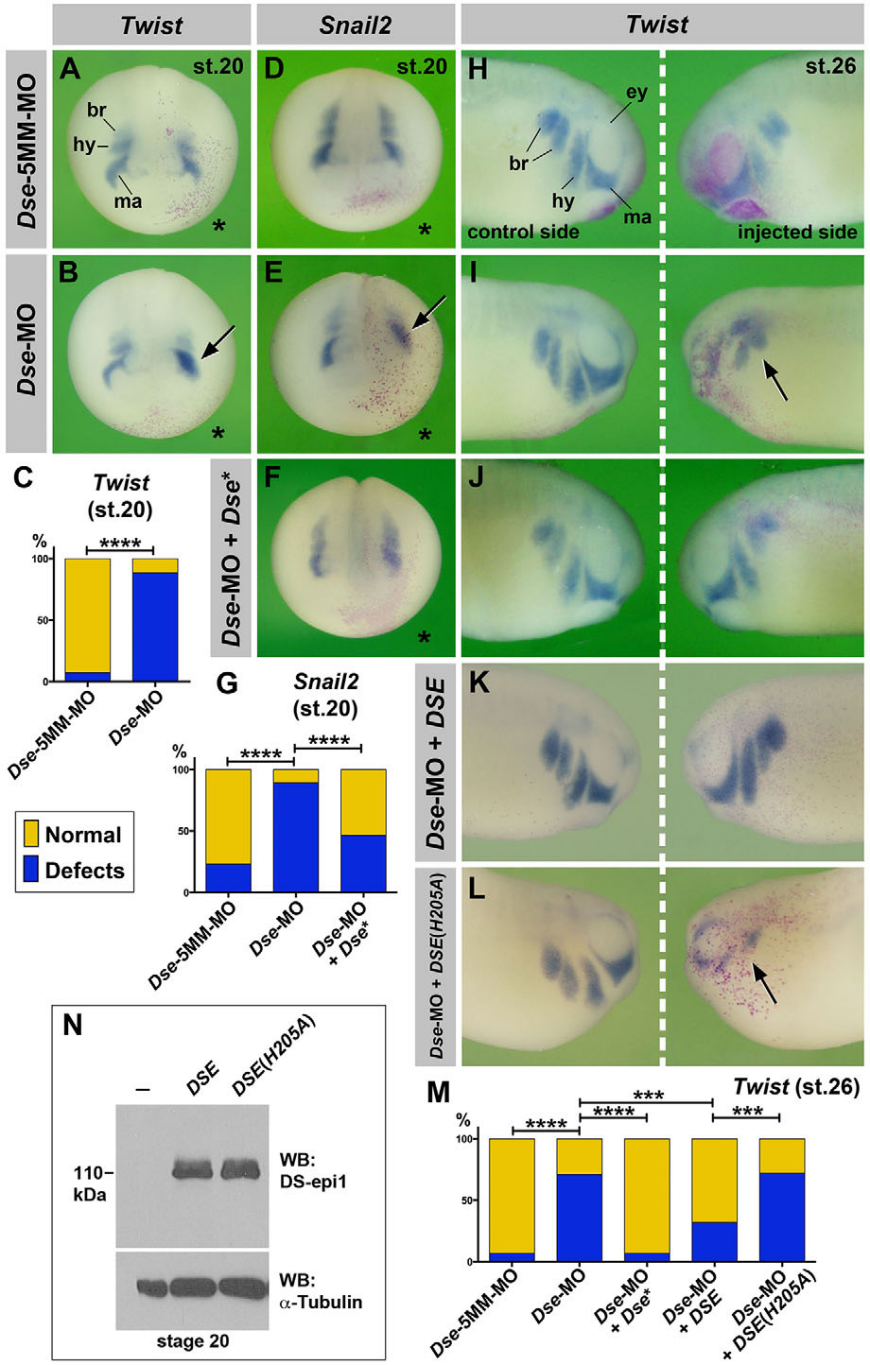
**DS-epi1 regulates CNC cell migration.** (A-G) Anterior view of late neurula embryos. The injected side is marked with a star. *Dse*-MO impairs the segregation of *Twist*^+^ and *Snail2*^+^ CNC cells (arrows). The effect is reversed by 250 pg Dse* mRNA. A quantification of the percentage of embryos with defects is shown in C and G. (H-M) Lateral view of tailbud embryos. *Dse*-MO leads to defective migration of *Twist*^+^ CNC cells (arrow) on the injected side, which is rescued by the co-injection of 250 pg Dse* mRNA and 25 pg pcDNA3/CTAP-*DSE* plasmid, but not 25 pg pcDNA3/CTAP-*DSE (H205A)* plasmid DNA. A quantification of the percentage of embryos with defects is shown in M. (N) Western blot analysis of lysates from embryos injected with 100 pg pcDNA3/CTAP-*DSE* or pcDNA3/CTAP-*DSE (H205A)* plasmid DNA and probed for DS-epi1. α-tubulin is a loading control. br, branchial segment; ey, eye; hy, hyoid segment; ma, mandibular segment. The proportion of examined embryos with the indicated phenotype was as follows: A, 15/16; B, 30/34; D, 41/46; E, 50/65; F, 20/37; H, 25/27; I, 31/41; J, 36/44; K, 27/40; and L, 14/18. ***P<0.005; ****P<0.0001 (Fisher's exact test with two-tailed *P*-value calculation).

At the late neurula stage, *Twist* and *Snail2* demarcate the mandibular, hyoid and branchial segments at the onset of CNC migration (Fig. 5A,D). DS-epi1 knockdown disturbed the segregation of CNC segments and shortened the early cell migration streams (Fig. 5B,C,E,G). In tailbud embryos, *Twist* and *Sox9* label migrated CNC cells around the eye vesicle (mandibular stream), and in the hyoid and branchial arches (Fig. 5H; Fig. S5D). *Dse*-MO restricted the ventral migration of the CNC cells (Fig. 5I,M; Fig. S5E,I) and decreased the *Rax* and *Sox9* expression in the eye and lens placode, respectively (Fig. S5C,E). The *Dse*-morphant migration defects were rescued by the co-injection of Dse* mRNA (Fig. 5F,G,J,M; Fig. S5F,I). Moreover, the injection of the human pcDNA3/CTAP-*DSE* expression plasmid restored normal CNC cell migration in the *Dse*-morphant embryos (Fig. 5K; Fig. S5G), whereas the catalytically inactive pcDNA3/CTAP*-DSE* (*H205A*) construct had no effect (Fig. 5L,M; Fig. S5H,I). Western blot analysis confirmed that pcDNA3/CTAP*-DSE* and pcDNA3/CTAP*-DSE* (*H205A*) (Pacheco et al., 2009a) in injected *Xenopus* embryos produced equal amounts of protein (Fig. 5N).

A TUNEL assay demonstrated that the *Dse*-MO had no detectable effect on apoptosis at the neurula or early tailbud stage; however, it induced cell death in advanced tailbud embryos (Fig. S6). The data indicate a distinct role of DS-epi1 in CNC cell migration.

### DS-epi1 has a tissue-autonomous role in cranial NC cell adhesion and migration

**Fig. 6.**
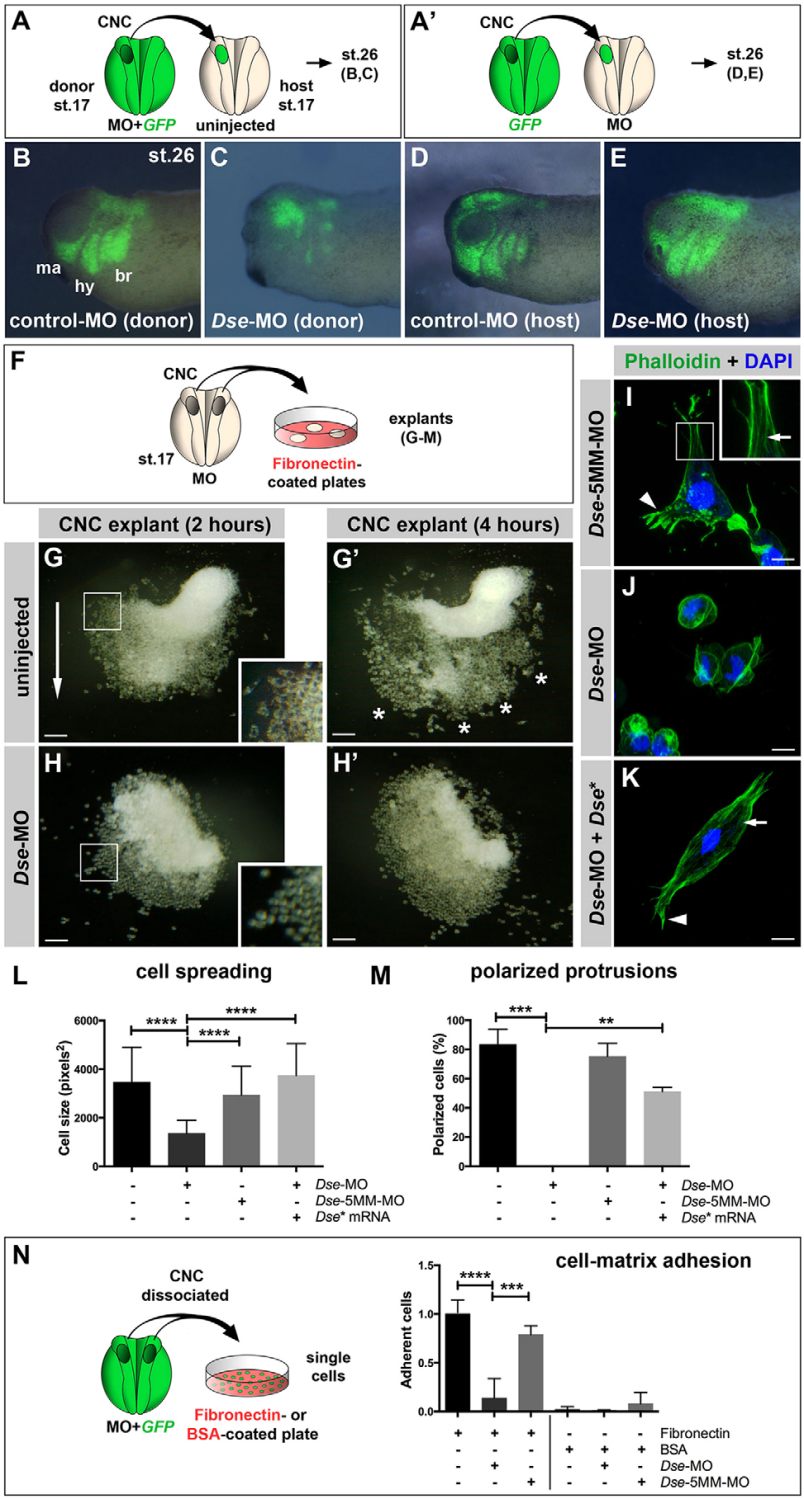
**DS-epi1 has a tissue-autonomous role in CNC cell migration, adherence to fibronectin and cell polarization.** (A,A′) Schemes for transplantation experiments. A CNC explant from an embryo injected with 300 pg *GFP* mRNA was homotypically grafted at stage 17. MOs were injected into the donor (A) or host embryo (A′). (B-E) Lateral view of embryos at stage 26. Grafted GFP^+^ CNC cells migrate ventrally when derived from control-MO-injected embryos (B); however, they do not properly migrate when derived from *Dse*-MO-injected embryos (C). br, branchial segment; hy, hyoid segment; ma, mandibular segment. The CNC cell migration was normal when the host embryo was injected with control-MO or *Dse*-MO (D,E). Three independent experiments were performed (*n*=3). (F) Scheme illustrating the culture of stage 17 morphant CNC explants on fibronectin-coated plates. (G,G′) At 2 h after plating (G), the control-MO-injected CNC explant exhibits collective cell migration in one direction (arrow). The inset shows a magnification of spread cells. After 4 h (G′), the cells migrate in distinct streams (asterisks). (H,H′) Cells of *Dse*-MO-injected CNC explants detach from each other and fail to adhere to the fibronectin substrate. The inset depicts a magnification of the spherical cells. (I-K) Confocal microscopy of fixed CNC cells after 5 h of explant culture on fibronectin. Phalloidin–Alexa-Fluor-488 and DAPI label F-actin and cell nuclei, respectively. The *Dse*-5MM-MO-injected control cell (I) exhibits lamellipodia at the leading edge (arrowhead) and stress fibers in the inner regions of the cell (arrow in inset). *Dse*-morphant cells (J) exhibit cortical networks of stress fibers and lack polarized protrusions. Co-injection of *Dse*-MO and 1 ng Dse* mRNA per embryo (K) restores the normal cytoskeleton and cell shape. (L,M) Quantification of cell spreading (L) and formation of polarized cell protrusions (M) in dissociated phalloidin-stained single cells from CNC explants following 5 h of culture on fibronectin. Cell spreading and polarized protrusions were quantified by calculating the cell size as the square number of pixels (ImageJ) and determining the percentage of cells with lamellipodia or filopodia, respectively. Uninjected and *Dse*-5MM-MO-injected explants exhibit a similar extent of cell spreading and formation of polarized protrusions. The reduction in the cell size and the lack of lamellipodia and filopodia are rescued by the co-injection of Dse* mRNA in *Dse*-morphant explants. A minimum of 100 cells per sample were evaluated in each experiment. Number of independent experiments (*n*≥3). Results are mean±s.d. (N) Cell–matrix adhesion of dissociated single CNC cells on fibronectin- or BSA-coated plates. Following the co-injection of MO and 300 pg *GFP* mRNA, CNC explants from stage 17 embryos were dissociated in Ca^2+^- and Mg^2+^-free medium and cultured for 45 min on fibronectin or BSA. The *Dse*-morphant cells exhibit decreased adhesion to fibronectin compared with the control and *Dse*-5MM-MO-injected cells. None of the analyzed cell samples exhibited significant cell adhesion to BSA. At least three independent experiments were performed for each sample (*n*≥3). Results are mean±s.d. The proportion of examined explants or cells with the indicated phenotype was as follows: B, 10/12; C, 11/13; D, 7/7; E, 9/9; G, 30/34; H, 26/28. Scale bars: 100 µm (G-H′); 10 µm (I-K). **P<0.01, ***P<0.001, ****P<0.0001 (one-way ANOVA multiple comparisons test with Tukey correction).

To investigate where the CS/DS chains affect cell migration, we performed transplantation experiments with green fluorescent protein (GFP) as a lineage tracer. First, we homotypically grafted CNC explants from embryos that were co-injected with a morpholino oligonucleotide and *GFP* mRNA into uninjected sibling hosts at stage 17 (Fig. 6A). Cells from the control-MO-injected CNC grafts migrated ventrally and formed well-orchestrated mandibular, hyoid and branchial streams (Fig. 6B). In contrast, the migration of the *Dse*-MO-injected CNC cells was disturbed and only a few labeled cells reached their correct destination (Fig. 6C). When *GFP*^+^ CNC cells were transplanted into control- and *Dse*-morphant host embryos (Fig. 6A′), their cell migration was normal (Fig. 6D,E). These findings suggest that DS-epi1 is needed in the CNC graft, but apparently not in surrounding tissue, which indicates a tissue-autonomous function of CS/DS PGs in CNC cell migration.

The adhesive extracellular matrix (ECM) glycoprotein fibronectin is ubiquitously distributed along NC migration pathways in the *Xenopus* embryo (Davidson et al., 2004). CNC undergoes cell migration on immobilized fibronectin substrates *in vitro* and exhibits a similar stream formation as demonstrated *in vivo* (Sadaghiani and Thiébaud, 1987; Alfandari et al., 2003). To further analyze the role of DS-epi1 in cell migratory behavior, we cultured CNC explants on fibronectin-coated plates (Fig. 6F). At 2 h after plating, one side of the uninjected CNC explants had expanded, and cells had spread on the substrate and migrated as a cohesive sheet (Fig. 6G). After 4 h, distinct segments started to form that were reminiscent of the mandibular, hyoid and branchial streams (Fig. 6G′). In contrast, the *Dse*-MO-injected CNC cells acquired a round morphology after 30 min, and did not subsequently spread or migrate (Fig. 6H,H′ and data not shown). To visualize the cytoskeleton, we performed phalloidin staining ∼5 h after plating, when cells typically started to individually migrate. The cells of *Dse*-5MM-MO-injected CNC explants exhibited F-actin^+^ stress fibers throughout the cytoplasm, in addition to lamellipodia and filopodia at their leading edges (Fig. 6I). *Dse*-morphant cells only formed cortical networks of stress fibers below the membrane (Fig. 6J). The degree of cell spreading and percentage of cells that formed polarized protrusions were similar between the *Dse-*5MM-MO-injected and uninjected CNC explants, whereas the *Dse*-morphant cells had a 60% smaller surface and no protrusions (Fig. 6L,M). Importantly, Dse* mRNA restored the normal cell size and shape in the *Dse*-MO-injected CNC explants (Fig. 6K-M), which suggests that DS-epi1 is required for cells to spread and form polarized cell protrusions.

To investigate cell–matrix adhesion, dissociated single GFP^+^ CNC cells were cultured for 45 min on fibronectin, and the percentage of the adherent cells was quantified (Fig. 6N). Approximately 90% of the *Dse*-MO-injected CNC cells lost adherence to fibronectin compared with the uninjected cells, whereas *Dse*-5MM-MO had no significant effect. As previously reported (Alfandari et al., 2003), CNC cells failed to attach to bovine serum albumin (BSA)-coated substrates. The observed defects were not a result of apoptosis because fluorescence-activated cell sorting (FACS) indicated that 2 h after the extraction, the CNC cells from the *Dse*-MO-injected embryos had >95% cell viability, which was comparable to that of the uninjected cells (Fig. S7A-C′). Moreover, the *Dse*-morphant CNC cells exhibited a normal cell cycle progression (Fig. S7D-F). At more than 4 h after the extraction, the *Dse*-MO-injected CNC cells did not have a lower survival rate compared with the uninjected controls (data not shown). Thus, our data on CNC cells indicate that IdoA in CS/DS is essential for cell adhesion and migration on fibronectin.

### Candidate CS/DS PGs in CNC cells

**Fig. 7.**
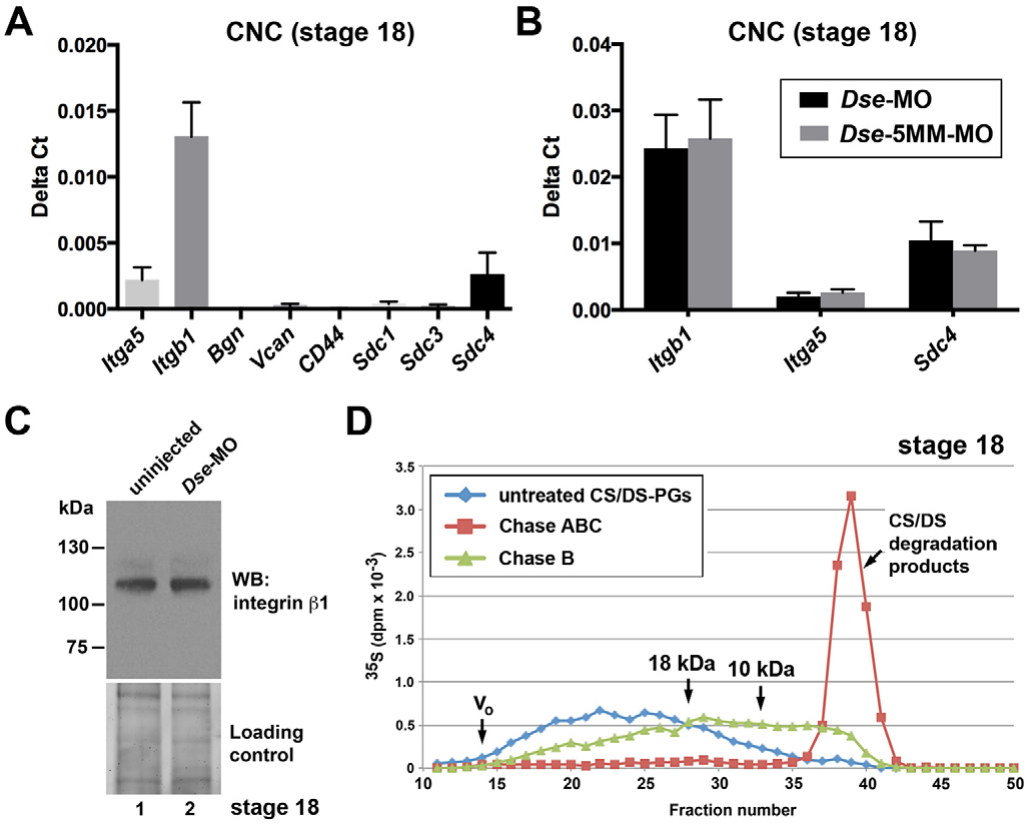
**CS/DS-PGs in CNC cells.** (A) qPCR analysis in uninjected CNC explants at stage 18. Note abundant expression of *Itga5*, *Itgb1* and *Sdc4*. Results are mean±s.d. from triplicates (*n*≥4 biological replicates). (B) *Dse*-MO does not differentially affect the mRNA levels of *Itga5*, *Itgb1* and *Sdc4* compared with *Dse*-5MM-MO. Results are mean±s.d. (*n*≥4 biological replicates). (C) *Dse*-MO does not reduce the protein amount of integrin β1 in explants enriched in neural crest and epidermis of stage 18 embryos. Western blotting was performed on a 7.5% Mini-Protean TGX Stain-free gel (Bio-Rad). The loading control was ascertained prior to blotting using the ChemiDoc Touch Imaging System. Resuts is representative of two independent experiments (*n*=2). (D) Metabolic labeling of PGs in stage 18 CNC explants. Note that Chase B partially degrades CS/DS PGs >18 kDa. The IdoA is a rare modification because the split chains are ∼10 kDa.

Based on the tissue-autonomous role of IdoA in CS/DS, we propose that DS-epi1 knockdown affects PGs that are synthesized in the CNC. The integrin α5β1 heterodimer (encoded by the *Itga5* and *Itgb1* genes), members of the syndecan (Sdc) family, Bgn, Vcan and CD44 have been presented as CS or CS/DS PGs, some of which have functions in cell adhesion (Thelin et al., 2013). In CNC explants of stage 18 embryos, qPCR indicated abundant mRNA levels of *Itga5*, *Itgb1* and *Sdc4*, whereas *Sdc1*, *Sdc3*, *Bgn*, *Vcan* and *CD44* were not expressed or were only expressed at very low levels (Fig. 7A). We did not identify changes in the *Itga5*, *Itgb1* or *Sdc4* mRNA levels in the *Dse*-morphant compared with the *Dse*-5MM-MO-injected control CNC cells (Fig. 7B). Moreover, western blot analysis demonstrated similar levels of integrin β1 protein in the DS-epi1^+^ and DS-epi1-deficient NC- and epidermis-enriched explants (Fig. 7C), which suggests that reduced IdoA levels do not alter the expression of integrins. In some mammalian cell types, it has been reported that the α5 and β1 integrin subunits (Veiga et al., 1997; Franco et al., 2009) and Sdc4 (Shworak et al., 1994; Deepa et al., 2004; Holmborn et al., 2012) are hybrid PGs that carry both CS and HS chains; however, it is not known whether they contain CS/DS chains. To investigate whether migratory CNC cells also synthesize IdoA in CS/DS chains, we isolated CNC explants at stage 18 and cultivated the cells *in vitro* in [^35^S]-containing medium for 24 h to label the PGs. Chase B degraded 47% of the labeled CS/DS PGs, which covered a wide range of molecular mass of over 18 kDa (Fig. 7D), and the size distribution of the degraded products indicated that isolated IdoA moieties are present in the native chains. The molecular mass of the IdoA-containing PGs is compatible with integrin α5β1 subunits (120-150 kDa) (Alfandari et al., 2003) and Sdc4 (40-250 kDa) (Gopal et al., 2010). However, we could not identify CS, CS/DS and HS chains on endogenous integrin α5 and β1 in *Xenopus* CNC tissue, as well as explants enriched in epidermis and neural crest (Fig. S8A,B). Moreover, we demonstrated that HS, but not CS and CS/DS, chains in Flag-tagged Sdc4 were produced in the mRNA-injected explants (Fig. S8C). In summary, integrin α5β1 and Sdc4 are expressed in CNC cells and do not appear to be decorated with CS or CS/DS chains in *Xenopus* embryos. These findings suggest that other not yet identified CS/DS PGs might mediate the DS-epi1-dependent CNC cell adhesion on fibronectin.

### *DSE* expression in NC-derived cancers

The involvement of DS-epi1 in NC migration in *Xenopus* embryos prompted us to investigate a potential link between *DSE* expression and tumorigenic properties in two human NC-derived cancers (Fig. S9). Gene set enrichment analysis of the expression datasets from both neuroblastoma and metastatic melanoma (*n*=498 and 44, respectively) demonstrated that *DSE* expression correlated with genes associated with EMT, mesenchymal and invasive phenotypes, as well as genes involved in metastasis. These findings indicate a potential role of DS-epi1 in NC-tumor development, which warrants further investigation.

## DISCUSSION

This study demonstrates, for the first time, an essential function of IdoA in early embryonic development and cell migration *in vivo*. The spatio-temporal expression of *Dse* in the *Xenopus* embryo suggested a role of DS-epi1 in ectoderm and NC development. The blockage of epimerase activity and IdoA biosynthesis upon the knockdown of DS-epi1 did not affect the allocation of neural and epidermal fates or the formation of NC progenitors. However, DS-epi1 deficiency altered the expression of neural-plate-border- and NC-specific transcription factors and decreased the extent of NC cell migration, which led to defects in craniofacial skeleton, melanocyte and dorsal fin formation. The functional links between DS-epi1 and EMT and between DS-epi1 cell adhesion on fibronectin, as established in this study for normal NC development, might have implications for neurocristopathies and cancer.

Our study demonstrates that, in *Xenopus* embryos, DS-epi1 is important for the formation of isolated IdoA moieties interspersed with unmodified GlcA residues. This result is noteworthy because previous findings in *Dse*-null mice (Maccarana et al., 2009) and a human patient with *Dse* loss-of-function (Müller et al., 2013) have suggested that DS-epi1 is mainly responsible for the biosynthesis of IdoA blocks. The amount and arrangement of IdoA residues in CS/DS chains depends on the protein core, tissue type and signaling status (Trowbridge and Gallo, 2002; Thelin et al., 2013). Our work comprises the first investigation to demonstrate *in vivo* that isolated IdoA residues change the biological properties of CS/DS PGs. Even a single IdoA is thought to render the CS/DS chain more flexible, which increases its ability to interact with growth factors and matrix components. For example, CS/DS chains isolated from embryonic pig brains contain sequences with a single IdoA residue that interacts with the growth factor pleiotrophin (Bao et al., 2005).

We note that despite the misregulation of NC-specific transcription factors, NC cells are formed in DS-epi1-deficient embryos. This finding is exemplified by the expression of *Twist*, which is initially reduced likely because of the concomitant downregulation of *Foxd3* expression (Sasai et al., 2001), but is subsequently restored in CNC cells at the post-neurula stage, possibly as a result of Wnt/β-catenin signaling, which induces NC cell fate and maintains *Twist* expression during migration (Chang and Hemmati-Brivanlou, 1998; Borchers et al., 2001). The downregulation of *Twist* and *Foxd3* expression in DS-epi1-morphant neurula embryos supports a function of DS-epi1 in EMT. Twist is a repressor of E-cadherin in *Xenopus* NC cells (Barriga et al., 2013). Low Twist and high E-cadherin cause a loss of polarized protrusions and a failure of cell–cell dissociation (Scarpa et al., 2015). Foxd3 promotes the EMT by modulating cell–cell adhesion in the chick embryo (Cheung et al., 2005; Fairchild et al., 2014). EMT inhibition might also explain the impaired segregation and reduced ventral migration of CNC cell streams following the knockdown of DS-epi-1.

A human esophagus squamous cell carcinoma cell line with short hairpin RNA (shRNA)-mediated DS-epi1 loss-of-function (Thelin et al., 2012) and cultured aortic smooth muscle cells from *Dse*^−/−^ mice (Bartolini et al., 2013) both have a reduced level of plasma membrane protrusions and an abnormal actin cytoskeleton, which changes their migration properties similar to the findings in CNC cells. Interestingly, DS-epi1 deficiency in these *in vitro* wound scratch and invasion assays leads to a larger cell size and increased speed, but decreased directionality, of cell migration. In comparison, DS-epi1 morphant CNC cells had a smaller cell size and did not migrate *in vitro* because of a failure in cell adhesion to fibronectin. These discrepancies might be a result of the different cell types and matrix components.

In loss-of-function experiments, DS-epi1 is not required for CNC cell survival at the neurula and early tailbud stage. However, the reduced cell anchorage of DS-epi1 morphant CNC cells might account for the increased apoptosis in advanced tailbud stage embryos. A positive role of DS-epi1 in the maintenance of CNC cells might be mediated by integrins, which convey survival signals in the CNC (Goh et al., 1997).

**Fig. 8.**
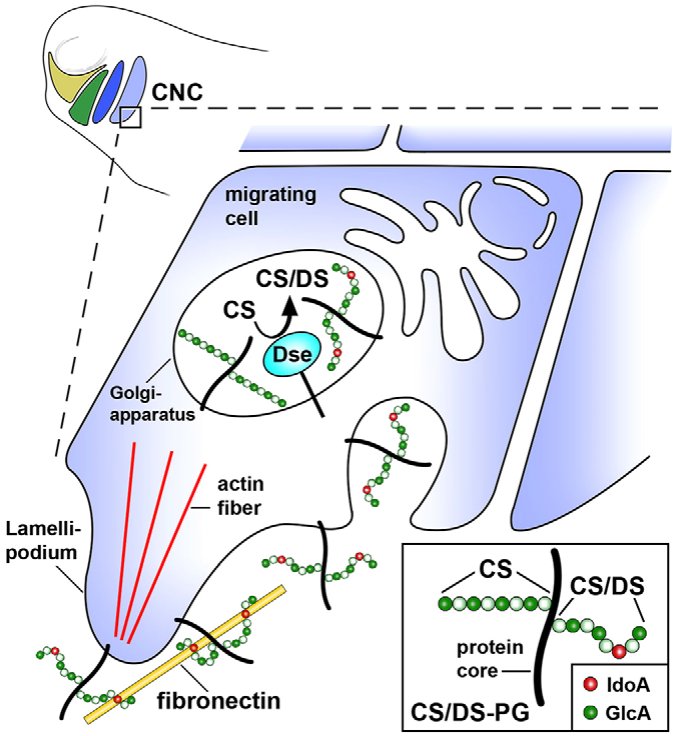
**Model for the stimulation of CNC cell migration by CS/DS PGs in a post-neurula embryo.** DS-epi1 converts GlcA into isolated IdoA residues on CS/DS PGs. The interaction between CS/DS PGs and extracellular fibronectin stimulates cytoskeletal rearrangement and polarized cell migration.

We suggest a model in which DS-epi1 converts selected GlcA residues into isolated IdoA on CS/DS PGs in *Xenopus* CNC cells (Fig. 8). Following interaction with fibronectin, CS/DS PGs lead to the rearrangement of the actin cytoskeleton and facilitate CNC cell adhesion and migration. In support of this model, we demonstrated in *Xenopus* CNC that (1) DS-epi1 is expressed and synthesizes IdoA on CS/DS-PGs, (2) DS-epi1 has a tissue-autonomous role in cell migration, and (3) DS-epi1 is required for the cells to adhere, spread and extend polarized cell processes on fibronectin. The integrin α5β1 heterodimer (Alfandari et al., 2003) and Sdc4 (Woods et al., 2000; Matthews et al., 2008) bind to distinct domains of fibronectin and are required for NC cell adhesion and migration. The interaction of GAG chains with fibronectin is crucial for the functions of integrin α5β1 in cell migration (Shworak et al., 1994) and Sdc4 in the proper organization of the actin cytoskeleton and cell adhesion (Gopal et al., 2010). Although co-expressed with *Dse* in the CNC at the onset of cell migration, *Itga5*, *Itgb1* and *Sdc4* mRNAs and the integrin β1 protein level are not altered in DS-epi1-deficient embryos. We did not identify CS or CS/DS chains in endogenous integrin α5β1, and overexpressed Flag–Sdc4 contained HS, but not CS chains, which indicates that the expression and GAG status of this integrin and Sdc4 are not affected by DS-epi1 in *Xenopus* embryos. A previous finding that purified DS binds to fibronectin (Saito and Munakata, 2007) supports a direct interaction between CS/DS PGs and this extracellular matrix component. Other mechanisms might exist to explain the role of IdoA in cell adhesion and migration. For example, CS/DS PGs might stimulate the cell surface localization of adhesion proteins or cooperate with fibronectin to activate integrins; these processes remain to be investigated.

MCEDS is the first disorder that has been discovered to specifically affect the biosynthesis of DS (Kosho et al., 2016). A defect in NC cells might contribute to the craniofacial phenotype in this condition. The features of MCEDS include hypoplasia of the jaw, high and/or cleft palate, midface skeletal anomaly, down-slanting palpedral fissues and malformed ears (Müller et al., 2013; Syx et al., 2015). These craniofacial anomalies also occur in congenital defects caused by a reduced amount or abnormal migration of NC cells into the first (mandibular) and second (hyoid) pharyngeal arch in Treacher Collins syndrome, Nager syndrome and Miller syndrome (Trainor and Andrews, 2013), which suggests a conserved role of DS in human NC development. The knockdown of DS-epi1 in *Xenopus* embryos phenocopies the craniofacial defects in humans. Our study in the *Xenopus* model suggests that an insufficient amount of NC cells migrating to their destinations might be a result of (1) a defect in the EMT, (2) an inability of NC cells to adhere to fibronectin, and (3) cell death as a consequence of anchorage loss of NC cells. Thus, our study suggests that MCEDS might add to the list of neurocristopathies. The identification of NC cells as a target of DS deficiency might help explain the other clinical criteria of MCEDS (Kosho, 2016). For example, it remains to be investigated whether the congenital heart defects in patients could be caused by an insufficient contribution of NC cells to the cardiac septum and valve formation, and whether recurrent subcutaneous infections in patients might reflect a compromised immune defense as a result of a reduced ability of NC cells to contribute to the thymus. In view of the multitude of cell types and tissues that NC cells give rise to (Le Douarin and Dupin, 2012), additional, new manifestations, e.g. in inner organs, might be identified that improve the diagnosis and etiology-based therapy of this currently intractable disorder.

Neurocristopathies also encompass NC-derived cancers, including neuroblastoma and malignant melanoma. Neuroblastoma is the most common solid cancer in the first year of life and is a leading cause of death in children with cancer. Malignant melanoma is the most aggressive skin cancer and the second most common cancer in individuals under the age of 50. Our study indicates that in NC-derived neuroblastoma and melanoma, *DSE* expression is correlated with gene sets for various migratory properties of cancer cells, including EMT, invasion and metastasis. During tumor progression, carcinoma cells acquire mesenchymal gene expression patterns, which results in migratory and invasive properties that enable the cells to disseminate through the body to produce metastatic tumors in distinct organs (Thiery et al., 2009; Tsai and Yang, 2013). Because of the heterogeneity of tumors (which have both epithelial and mesenchymal cells), it has been difficult to investigate the EMT in cancer development *in vivo* (Tsuji et al., 2009). Our *Xenopus* NC model has established a functional link between DS-epi1 and cell migration. The correlation between DS-epi1 expression and EMT in NC-derived cancers suggests a possible involvement of DS in metastatic diseases that remains to be investigated.

The demonstration that DS is important for cell migration *in vivo* might shed light on other processes that are dependent on both DS (Syx et al., 2015) and fibronectin (Muro et al., 2003; Castelletti et al., 2008), such as skin wound healing and kidney function. These processes might also involve a functional link between DS and fibronectin in cell adhesion, as proposed in the present study.

## MATERIALS AND METHODS

### Constructs and microinjection

A full-length cDNA clone of *X. laevis Dse.S* in pCS105 (ID: XL487g09ex) that contained 251 nucleotides of the 5′ untranslated region (UTR), the open reading frame (ORF) and 680 nucleotides of the 3′UTR was obtained from the Osada/Taira NBRP *Xenopus* ANE library (Osada et al., 2003) and completely sequenced (GenBank accession number KU877109). To obtain pCS2-Dse*, the ORF of *Dse.S* was point-mutated by performing PCR and subcloned into pCS2, which resulted in seven nucleotide mismatches in the *Dse*-MO target sequence in positions −2, −1, +6, +9, +12, +15 and +18 relative to the translation start site. A full-length cDNA clone of *X. laevis Dsel.L* with 93 nucleotides of the 5′UTR, the ORF and 22 nucleotides of the 3′UTR (GenBank accession number KU877110) was generated by RT-PCR and subcloned into the pCR-XL-TOPO vector (Thermo Fisher Scientific), using total RNA from the head of stage 28 embryos and primers based on a corresponding Xenbase sequence entry.

Sense RNAs for microinjection were synthesized using the mMessage Machine kit (Ambion). cDNA plasmids were linearized and transcribed as follows: pCS105-*Dse* (BstX1, Sp6), pCS2-Dse* (NotI, Sp6), pCS2-*nLacZ*, (NotI, Sp6), pCS2-*Flag-Sdc4* (NotI, Sp6; Muñoz et al., 2006) and pCMT-*eGFP* (NotI, Sp6; a kind gift from Eric Bellefroid, Université Libre de Bruxelles, Belgium). The *Dse*-MO (5′-GCTCCCCGAGTGTGAGTCCTCATTG-3′), *Dse*-5MM-MO (5′-GCTaCCCcAGTcTGAGTaCTaATTG-3′; lowercase letters represent mismatches), *Dsel*-MO (5′-ATGGTCCATTAGGAGAATAGTCAGT-3′) and standard control-MO (5′-CCTCTTACCTCAGTTACAATTTATA-3′) were obtained from Gene Tools LLC. Unless otherwise stated, mRNAs, DNAs and MOs were injected into the animal pole of all blastomeres at the two- or four-cell stage. A total of 45 ng MOs was injected per embryo. For single injections, a quarter of the MO amount with 125 pg *nlacZ* mRNA as a lineage tracer was used.

### Embryo manipulations and histological staining

All *Xenopus laevis* experiments reported in this study were approved by the Lund/Malmö regional ethical committee (M140-14). The embryos were prepared, cultured and analyzed by Red-Gal staining, whole-mount *in situ* hybridization and TUNEL assay as described previously (Pera et al., 2015). For histological staining, embryos were fixed in Bouin's solution, dehydrated, embedded in paraffin, sectioned at 10 μm, dewaxed and stained with hematoxylin and eosin. For cartilage staining, tadpoles were fixed in MEMFA (0.1 M MOPS, 2 mM EGTA, 1 mM MgSO_4_, 3.7% formaldehyde, pH 7.4), dehydrated, and stained in Alcian Blue solution; the facial skeleton was dissected after rehydration in 80% glycerol in 2% KOH.

### Preparation of explants and transplantation

Explants enriched in neural crest and epidermis were prepared by removing the chorion membrane with forceps, hemi-sectioning stage 18 embryos, and cutting off the neural epithelium and yolk with a surgical blade (Swann-Morton 10, REF 0301) on 1% agarose plates in MBS.

CNC explants from the embryos injected into the animal hemisphere with *eGFP* mRNA at stage 17 were dissected and homotypically grafted into host embryos as previously described (Borchers et al., 2000) with the following modifications: the dissection and grafting of the CNC explants were performed on 1% agarose in MBS. At ∼30 min after grafting, the embryo was transferred to a clean dish filled with 0.1× MBS and incubated at 17°C. At stage 26, the migration pattern of the transplanted CNC cells was monitored with a fluorescence stereomicroscope (Leica MZFLIII) and documented with a digital camera (Nikon DS-Fi1c).

### CNC migration and adhesion assays

Substrates, including human plasma fibronectin (Millipore) and BSA (Sigma), were diluted at 10 µg/ml in PBS and coated on plastic at 4°C overnight. The migration assay with CNC explants on fibronectin-coated plastic dishes was performed as previously described (DeSimone et al., 2005). For the adhesion assay, fluorescently labeled CNC explants from the embryos injected into the animal hemisphere with *eGFP* mRNA were incubated in Ca^2+^- and Mg^2+^-free MBS for 1 h at 12°C to dissociate the cells (Alfandari et al., 2003). The single CNC cells were subsequently plated in 96-well plates coated with fibronectin or BSA and allowed to attach for 45 min at room temperature. Photographs were taken with a fluorescence microscope shortly before and after gently pipetting the medium up and down, and the attached cells were counted.

### Cytoskeletal staining

CNC explants were plated on fibronectin-coated cover glass slides (100 µg/ml). Following 5 h of culture, the explants were fixed in 4% formaldehyde in PBS for 1 h and blocked for 1 h in PBS with 1% BSA. The explants were incubated for 20 min with phalloidin–Alexa-Fluor-488 (1:100 in PBS, Life Technologies, A12379). Cover slides were mounted with Fluorescent Mounting Medium (Dako) that contained 4′,6-diamidino-2-phenylindole (DAPI), and the staining was monitored with a Zeiss LSM 780 confocal microscope.

### RT-PCR and qPCR analysis

Total RNA was extracted with TRIzol^®^ reagent (Ambion-Life Technologies). cDNA synthesis was performed using Reverse Transcriptase AMV (Roche) for whole embryos and SuperScript^®^ II Reverse Transcriptase (Invitrogen Life Technologies) for CNC explants. RT-PCR was performed as previously reported (Hou et al., 2007). qPCR was performed on ten CNC explants per sample using the Fast SYBR^®^ Green Master Mix (Ambion Life Technologies) and the StepOne™ Real-Time PCR System (Life Technologies). The relative expression was evaluated using the ΔCt method with the housekeeping gene *eEF1A1* as the control. The primers are listed in Tables S1 and S2.

### Epimerase assay

Embryos (*n*=250 to investigate the activity at different stages; *n*=50 to investigate the effect of morpholinos) were lysed in 1 ml PBS, 1% Triton X-100, 1 mM DTT, 1 mM EDTA and protease inhibitors (Complete Mini; Roche, 04693124001). Cleared supernatant was exchanged with dialysis buffer (20 mM MES, pH 5.5 at 37°C, 10% glycerol, 0.5 mM EDTA, 0.1% Triton X-100, 1 mM PMSF and 1 mM DTT) using PD10 columns (GE Healthcare). The protein content was measured by performing a Bradford assay (Bio-Rad), using bovine serum albumin (BSA) as the standard. Equal amounts of protein were assayed in a final 100 µl reaction buffer that was composed of dialysis buffer as previously described, which contained 2 mM MnCl_2_, 0.5% NP-40, 100 µg BSA and 30,000 dpm of the labeled chondroitin substrate ([5-^3^H]defructosylated, K4 prepared as previously described; Hannesson et al., 1996). Incubations were conducted for 20 h at 37°C, and the released tritium was quantified as previously described (Pacheco et al., 2009a). During the assay, it was determined that the inhibitory components in the lysates varied in the embryos of different developmental stages. To remove these inhibitions and to increase the sensitivity of the assay, in particular during the early stages, the lysates were enriched in epimerase activity by purification on Red-Sepharose gel (Pacheco et al., 2009a; Gustafsson et al., 2014). A total of 2 mg of lysates in 1 ml 60 mM MES pH 6.5 and 100 mM NaCl were prepared and batch-incubated overnight at 4°C with 30 µl Red-Sepharose gel (GE Healthcare). The gel was washed with incubation buffer, and the epimerases were eluted with 200 µl dialysis buffer, 2 M NaCl and 1 mg/ml BSA as the carrier. The samples were desalted on membranes by using dialysis buffer, and 50 µl of the preparation was subsequently assayed.

### Lyase treatment

Proteoglycans were treated for 2 h at 37°C with 10 mIU chondroitinase ABC (Sigma) in 20 µl ammonium acetate pH 8.0 or 5 mIU chondroitinase B (R&D Systems) in 20 µl 50 mM Tris-HCl pH 7.5, 50 mM NaCl, 4 mM CaCl_2_ or a mixture of 2 mIU each of heparinases I, II and III in 20 μl 40 mM sodium acetate, pH 7.0, and 2 mM calcium acetate (heparinases were prepared in-house, purified from *E.coli*, stably singularly transfected with the pET-15b vector that contained heparinase I or vector pET-19b that contained heparinase II or III, a kind gift from Jian Liu, University of North Carolina, NC).

### Metabolic labeling of proteoglycans and GAG analysis

[^35^S]sulfate (Perkin-Elmer; 600 nCi in 25 nl/embryo) was injected into the blastocoel of stage 9 embryos, and PGs were purified at stage 22. The PGs were extracted in 4 M guanidine, 50 mM sodium acetate, pH 5.8, 0.1% Triton X-100, 1 mM EDTA, 10 mM *N*-ethylmaleimide and protease inhibitors. The extraction buffer was exchanged to 6 M urea, pH 5.5, 0.1% Triton X-100, 1 mM EDTA, 0.2 M NaCl and protease inhibitors, and the PGs were anion-exchange purified using a DE52 gel. The purified labeled PGs, desalted with water, were treated with lyases as described above. The samples were subsequently deaminated in nitrous acid at pH 1.5 to degrade heparan sulfate (Shively and Conrad, 1976).

Proteoglycan labeling using dissociated cells of ten isolated CNC explants, which was adapted in consideration of the low number of available cells, was performed by changing the culture medium with a fresh medium that contained 2 mCi [^35^S]sulfate/ml and 10 µM sodium sulfate, and cultivating the cells for 24 h at 17°C. The culture medium was removed, and the cells were washed and solubilized with the urea-containing buffer as described above, followed by anion-exchange purification and HS degradation as described above. The CS/DS PGs were re-isolated by anion-exchange purification and desalted. Lyase treatment was performed as described above.

The control and lyase-treated, labeled PGs were analyzed by SDS-PAGE as previously described (Pacheco et al., 2009b); alternatively, they were applied to a size-permeation Superose 6 column (GE Healthcare) and run in the urea-containing buffer as previously described, and the eluted fractions were counted by β-scintillation.

### Immunoblotting of DS-epi1

Ten embryos were lysed in modified Barth solution (MBS) (88 mM NaCl, 1 mM KCl, 0.7 mM CaCl_2_, 1 mM MgSO_4_, 2.5 mM NaHCO_3_, 5 mM HEPES, pH 7.4), 1% Triton X-100, 5 mM EDTA and proteinase inhibitors for 30 min on ice and centrifuged at 10,000 ***g*** for 30 min at 4°C. The protein content was measured using the Bradford method (Bio-Rad), with BSA as the standard. Equal protein amounts were separated on 8% SDS-PAGE, and immunoblotting was performed using an immunopurified anti-DS-epi1 antibody (1 μg/ml) (Pacheco et al., 2009a).

### Immunoprecipitation of integrin α5

Twenty CNC explants from stage 18 embryos were incubated in Ca^2+^- and Mg^2+^-free MBS for 1 h at 12°C, and the dissociated cells were labeled with 500 μl of a 2 mg/ml sulfo-NHS-LC-biotin solution (Pierce, 21335) for 20 min at 15°C. After three washes with 100 mM of a glycine solution, the cells were lysed in MBS, 1% Triton X-100, 5 mM EDTA and proteinase inhibitors. Following centrifugation at 12,000 ***g*** for 30 min at 4°C, immunoprecipitation was performed overnight with 10 μg of anti-Intα5 antibody (P8D4, a kind gift from Dominique Alfandari, University of Massachusetts, MA) loaded on Protein A/G agarose beads (Invitrogen, 20423). The beads were washed three times in Tris-buffered saline (TBS) that contained 1% Triton X-100 and once in TBS, treated with lyases and ultimately eluted in 2× non-reducing Laemmli Buffer. Proteins were separated by 8% SDS-PAGE, transferred onto nitrocellulose and detected with horseradish peroxide (HRP)-conjugated streptavidin (Invitrogen, 1:75,000).

### Immunoblotting of integrin β1 and Flag-Sdc4

Cleared lysates from the epidermis-enriched explants of 12 uninjected embryos were lysed in MBS, 1% Triton X-100, 5 mM EDTA and proteinase inhibitors for 30 min on ice, centrifuged at 10,000 ***g*** for 30 min at 4°C and dialyzed with 10 mM sodium acetate pH 7.0. The protein content was measured using the Bradford method (Bio-Rad), with BSA as a standard. Equal protein amounts were treated with lyases as described above.

Epidermal explant lysates from embryos injected into the animal hemisphere with *Flag–Sdc4* mRNA and uninjected controls were diluted 1:3 in 9 M urea, pH 5.5, 1 mM EDTA, 0.1% Triton X-100, 0.2M NaCl and protease inhibitors, and were anion-exchange purified using 20 μl of DE52 gel. The released proteoglycans in 2 M ammonium bicarbonate, 0.05% Triton and BSA, which was added as a carrier, were diluted 1:10 in water and lyophilized. Equal parts of the resuspended PGs were treated with lyases. Then, equal protein amounts were separated by 8% SDS-PAGE, and immunoblotting was performed using a monoclonal anti-Intβ1 antibody (8C8, 1:140, DSHB) and HRP-conjugated anti-Flag antibody (1:1000, Sigma, A8592).

### Fluorescence-activated cell sorting

To assess cell viability, dissociated cells from 20 CNC explants were incubated with 10 μg of propidium iodide (PI) and analyzed ∼2 h after extraction by performing FACS, with BD FACSCalibur™ and BD cellQuest™ pro software for acquisition. Analyses were performed with Flojow version X. To monitor the cell cycle, dissociated CNC cells were fixed with ethanol at 4°C overnight. The cells were subsequently washed and incubated with 10 μg of RNase A for 15 min. Prior to the analyses, the cells were incubated with 10 μg PI.

### Gene expression analyses

Two publically available datasets, which comprised 498 neuroblastoma tumors (Wang et al., 2014) and 44 metastatic melanomas (Bogunovic et al., 2009), respectively, were obtained from the NCBI GEO website (http://www.ncbi.nlm.nih.gov/geo/; accession numbers GSE62564 and GSE19234, respectively). Ranked gene lists of the correlations between all genes and DSE expression were produced using Pearson correlations. A gene set enrichment analysis (GSEA; Subramanian et al., 2005) was subsequently performed on the ranked gene lists using the c2.v5 curated gene set collection available from the Molecular Signatures Database (MsigDB; broad.mit.edu/gsea/msigdb).

## References

[DMM024661C1] Alfandari D., Cousin H., Gaultier A., Hoffstrom B. G. and DeSimone D. W. (2003). Integrin α5β1 supports the migration of Xenopus cranial neural crest on fibronectin. *Dev. Biol.* 260, 449-464. 10.1016/S0012-1606(03)00277-X 12921745

[DMM024661C3] Bao X., Muramatsu T. and Sugahara K. (2005). Demonstration of the pleiotrophin-binding oligosaccharide sequences isolated from chondroitin sulfate/dermatan sulfate hybrid chains of embryonic pig brains. *J. Biol. Chem.* 280, 35318-35328. 10.1074/jbc.M507304200 16120610

[DMM024661C4] Barriga E. H., Maxwell P. H., Reyes A. E. and Mayor R. (2013). The hypoxia factor Hif-1α controls neural crest chemotaxis and epithelial to mesenchymal transition. *J. Cell Biol.* 201, 759-776. 10.1083/jcb.201212100 23712262 PMC3664719

[DMM024661C5] Bartolini B., Thelin M. A., Rauch U., Feinstein R., Oldberg Å., Malmström A. and Maccarana M. (2012). Mouse development is not obviously affected by the absence of dermatan sulfate epimerase 2 in spite of a modified brain dermatan sulfate composition. *Glycobiology* 22, 1007-1016. 10.1093/glycob/cws065 22496542

[DMM024661C6] Bartolini B., Thelin M. A., Svensson L., Ghiselli G., van Kuppevelt T. H., Malmström A. and Maccarana M. (2013). Iduronic acid in chondroitin/dermatan sulfate affects directional migration of aortic smooth muscle cells. *PLoS ONE* 8, e66704 10.1371/journal.pone.0066704 23843960 PMC3699603

[DMM024661C7] Bogunovic D., O'Neill D. W., Belitskaya-Levy I., Vacic V., Yu Y.-L., Adams S., Darvishian F., Berman R., Shapiro R. and Pavlick A. C. (2009). Immune profile and mitotic index of metastatic melanoma lesions enhance clinical staging in predicting patient survival. *Proc. Natl. Acad. Sci. USA* 106, 20429-20434. 10.1073/pnas.0905139106 19915147 PMC2787158

[DMM024661C8] Borchers A., Epperlein H.-H. and Wedlich D. (2000). An assay system to study migratory behavior of cranial neural crest cells in Xenopus. *Dev. Genes Evol.* 210, 217-222. 10.1007/s004270050307 11180825

[DMM024661C9] Borchers A., David R. and Wedlich D. (2001). Xenopus cadherin-11 restrains cranial neural crest migration and influences neural crest specification. *Development* 128, 3049-3060. 11688555 10.1242/dev.128.16.3049

[DMM024661C10] Casini P., Ori M., Avenoso A., D'Ascola A., Traina P., Mattina W., Perris R., Campo G. M., Calatroni A. and Nardi I. (2008). Identification and gene expression of versican during early development of Xenopus. *Int. J. Dev. Biol.* 52, 993-998. 10.1387/ijdb.082582pc 18956330

[DMM024661C11] Castelletti F., Donadelli R., Banterla F., Hildebrandt F., Zipfel P. F., Bresin E., Otto E., Skerka C., Renieri A. and Todeschini M. (2008). Mutations in FN1 cause glomerulopathy with fibronectin deposits. *Proc. Natl. Acad. Sci. USA* 105, 2538-2543. 10.1073/pnas.0707730105 18268355 PMC2268172

[DMM024661C12] Chang C. and Hemmati-Brivanlou A. (1998). Neural crest induction by Xwnt7B in Xenopus. *Dev. Biol.* 194, 129-134. 10.1006/dbio.1997.8820 9473337

[DMM024661C13] Cheung M., Chaboissier M.-C., Mynett A., Hirst E., Schedl A. and Briscoe J. (2005). The transcriptional control of trunk neural crest induction, survival, and delamination. *Dev. Cell* 8, 179-192. 10.1016/j.devcel.2004.12.010 15691760

[DMM024661C14] Davidson L. A., Keller R. and DeSimone D. W. (2004). Assembly and remodeling of the fibrillar fibronectin extracellular matrix during gastrulation and neurulation in Xenopus laevis. *Dev. Dyn.* 231, 888-895. 10.1002/dvdy.20217 15517579

[DMM024661C15] Deepa S. S., Yamada S., Zako M., Goldberger O. and Sugahara K. (2004). Chondroitin sulfate chains on syndecan-1 and syndecan-4 from normal murine mammary gland epithelial cells are structurally and functionally distinct and cooperate with heparan sulfate chains to bind growth factors: a novel function to control binding of midkine, pleiotrophin, and basic fibroblast growth factor. *J. Biol. Chem.* 279, 37368-37376. 10.1074/jbc.M403031200 15226297

[DMM024661C16] DeSimone D. W., Davidson L., Marsden M. and Alfandari D. (2005). The Xenopus embryo as a model system for studies of cell migration. *Cell Migr.* 294, 235-245. 10.1385/1-59259-860-9:235 15576916

[DMM024661C17] Fairchild C. L., Conway J. P., Schiffmacher A. T., Taneyhill L. A. and Gammill L. S. (2014). FoxD3 regulates cranial neural crest EMT via downregulation of tetraspanin18 independent of its functions during neural crest formation. *Mech. Dev.* 132, 1-12. 10.1016/j.mod.2014.02.004 24582980 PMC4001935

[DMM024661C18] Franco C. R. C., Trindade E. S., Rocha H. A. O., da Silveira R. B., Paludo K. S., Chammas R., Veiga S. S., Nader H. B. and Dietrich C. P. (2009). Glycosaminoglycan chains from alpha5beta1 integrin are involved in fibronectin-dependent cell migration. *Biochem. Cell Biol.* 87, 677-686. 10.1139/O09-047 19767830

[DMM024661C20] Goh K. L., Yang J. T. and Hynes R. O. (1997). Mesodermal defects and cranial neural crest apoptosis in alpha5 integrin-null embryos. *Development* 124, 4309-4319. 9334279 10.1242/dev.124.21.4309

[DMM024661C21] Gopal S., Bober A., Whiteford J. R., Multhaupt H. A. B., Yoneda A. and Couchman J. R. (2010). Heparan sulfate chain valency controls syndecan-4 function in cell adhesion. *J. Biol. Chem.* 285, 14247-14258. 10.1074/jbc.M109.056945 20154082 PMC2863221

[DMM024661C22] Gorlin R. J., Cohen M. M. and Hennekam R. C. (1990). *Syndromes of the Head and Neck*. New York: Oxford University Press.

[DMM024661C23] Gustafsson R., Stachtea X., Maccarana M., Grottling E., Eklund E., Malmström A. and Oldberg Å. (2014). Dermatan sulfate epimerase 1 deficient mice as a model for human abdominal wall defects. *Birth Defects Res. Part A Clin. Mol. Terat.* 100, 712-720. 10.1002/bdra.23300 PMC4233991 25186462

[DMM024661C68] Hannesson H. H., Hagner-McWhirter A., Tiedemann K., Lindahl U. and Malmström A. (1996). Defructosylated Escherichia coli K4 capsular polysaccharide as a substrate for the D-glucuronyl C-5 epimerase, and an indication of a two-base reaction mechanism. *Biochem. J.* 313, 589-596. 10.1042/bj3130589 8573097 PMC1216948

[DMM024661C24] Holmborn K., Habicher J., Kasza Z., Eriksson A. S., Filipek-Gorniok B., Gopal S., Couchman J. R., Ahlberg P. E., Wiweger M. and Spillmann D. (2012). On the roles and regulation of chondroitin sulfate and heparan sulfate in zebrafish pharyngeal cartilage morphogenesis. *J. Biol. Chem.* 287, 33905-33916. 10.1074/jbc.M112.401646 22869369 PMC3460485

[DMM024661C25] Hou S., Maccarana M., Min T. H., Strate I. and Pera E. M. (2007). The secreted serine protease xHtrA1 stimulates long-range FGF signaling in the early Xenopus embryo. *Dev. Cell* 13, 226-241. 10.1016/j.devcel.2007.07.001 17681134

[DMM024661C26] Iozzo R. V. and Schaefer L. (2015). Proteoglycan form and function: a comprehensive nomenclature of proteoglycans. *Matrix Biol.* 42, 11-55. 10.1016/j.matbio.2015.02.003 25701227 PMC4859157

[DMM024661C27] Kosho T. (2016). CHST14/D4ST1 deficiency: a new form of Ehlers-Danlos syndrome. *Pediatr. Int.* 58, 88-99. 10.1111/ped.12878 26646600

[DMM024661C28] Le Douarin N. M. and Dupin E. (2012). The neural crest in vertebrate evolution. *Curr. Opin. Genet. Dev.* 22, 381-389. 10.1016/j.gde.2012.06.001 22770944

[DMM024661C29] Maccarana M., Olander B., Malmström J., Tiedemann K., Aebersold R., Lindahl U., Li J.-P. and Malmström A. (2006). Biosynthesis of dermatan sulfate: chondroitin-glucuronate C5-epimerase is identical to SART2. *J. Biol. Chem.* 281, 11560-11568. 10.1074/jbc.M513373200 16505484

[DMM024661C30] Maccarana M., Kalamajski S., Kongsgaard M., Magnusson S. P., Oldberg Å. and Malmström A. (2009). Dermatan sulfate epimerase 1-deficient mice have reduced content and changed distribution of iduronic acids in dermatan sulfate and an altered collagen structure in skin. *Mol. Cell. Biol.* 29, 5517-5528. 10.1128/MCB.00430-09 19687302 PMC2756890

[DMM024661C31] Matthews H. K., Marchant L., Carmona-Fontaine C., Kuriyama S., Larraín J., Holt M. R., Parsons M. and Mayor R. (2008). Directional migration of neural crest cells in vivo is regulated by Syndecan-4/Rac1 and non-canonical Wnt signaling/RhoA. *Development* 135, 1771-1780. 10.1242/dev.017350 18403410

[DMM024661C32] Mayor R. and Theveneau E. (2013). The neural crest. *Development* 140, 2247-2251. 10.1242/dev.091751 23674598

[DMM024661C33] Moreno M., Muñoz R., Aroca F., Labarca M., Brandan E. and Larraín J. (2005). Biglycan is a new extracellular component of the Chordin–BMP4 signaling pathway. *EMBO J.* 24, 1397-1405. 10.1038/sj.emboj.7600615 15775969 PMC1142540

[DMM024661C34] Müller T., Mizumoto S., Suresh I., Komatsu Y., Vodopiutz J., Dundar M., Straub V., Lingenhel A., Melmer A. and Lechner S. (2013). Loss of dermatan sulfate epimerase (DSE) function results in musculocontractural Ehlers–Danlos syndrome. *Hum. Mol. Genet.* 22, 3761-3772. 10.1093/hmg/ddt227 23704329

[DMM024661C69] Muñoz R., Moreno M., Oliva C., Orbenes C. and Larraín J. (2006). Syndecan-4 regulates non-canonical Wnt signalling and is essential for convergent and extension movements in Xenopus embryos. *Nat. Cell Biol.* 8, 492-500. 10.1038/ncb1399 16604063

[DMM024661C35] Muro A. F., Chauhan A. K., Gajovic S., Iaconcig A., Porro F., Stanta G. and Baralle F. E. (2003). Regulated splicing of the fibronectin EDA exon is essential for proper skin wound healing and normal lifespan. *J. Cell Biol.* 162, 149-160. 10.1083/jcb.200212079 12847088 PMC2172721

[DMM024661C36] Nakao M., Shichijo S., Imaizumi T., Inoue Y., Matsunaga K., Yamada A., Kikuchi M., Tsuda N., Ohta K. and Takamori S. (2000). Identification of a gene coding for a new squamous cell carcinoma antigen recognized by the CTL. *J. Immun.* 164, 2565-2574. 10.4049/jimmunol.164.5.2565 10679095

[DMM024661C37] Osada S.-I., Ohmori S.-Y. and Taira M. (2003). XMAN1, an inner nuclear membrane protein, antagonizes BMP signaling by interacting with Smad1 in Xenopus embryos. *Development* 130, 1783-1794. 10.1242/dev.00401 12642484

[DMM024661C38] Pacheco B., Maccarana M., Goodlett D. R., Malmström A. and Malmström L. (2009a). Identification of the active site of DS-epimerase 1 and requirement of N-glycosylation for enzyme function. *J. Biol. Chem.* 284, 1741-1747. 10.1074/jbc.M805479200 19004833

[DMM024661C39] Pacheco B., Malmström A. and Maccarana M. (2009b). Two dermatan sulfate epimerases form iduronic acid domains in dermatan sulfate. *J. Biol. Chem.* 284, 9788-9795. 10.1074/jbc.M809339200 19188366 PMC2665100

[DMM024661C40] Pera E. M., Acosta H., Gouignard N. and Climent M. (2015). Whole-mount in situ hybridization and immunohistochemistry in Xenopus embryos. *In Situ Hybridization Methods* 99, 151-167. 10.1007/978-1-4939-2303-8_8

[DMM024661C41] Powell D. R., Blasky A. J., Britt S. G. and Artinger K. B. (2013). Riding the crest of the wave: parallels between the neural crest and cancer in epithelial-to-mesenchymal transition and migration. *Wiley Interdiscip. Rev. Syst. Biol. Med.* 5, 511-522. 10.1002/wsbm.1224 23576382 PMC3739939

[DMM024661C42] Sadaghiani B. and Thiébaud C. H. (1987). Neural crest development in the Xenopus laevis embryo, studied by interspecific transplantation and scanning electron microscopy. *Dev. Biol.* 124, 91-110. 10.1016/0012-1606(87)90463-5 3666314

[DMM024661C43] Saito A. and Munakata H. (2007). Analysis of plasma proteins that bind to glycosaminoglycans. *Biochim. Biophys. Acta* 1770, 241-246. 10.1016/j.bbagen.2006.10.015 17178194

[DMM024661C44] Sasai N., Mizuseki K. and Sasai Y. (2001). Requirement of FoxD3-class signaling for neural crest determination in Xenopus. *Development* 128, 2525-2536. 11493569 10.1242/dev.128.13.2525

[DMM024661C45] Scarpa E., Szabó A., Bibonne A., Theveneau E., Parsons M. and Mayor R. (2015). Cadherin switch during EMT in neural crest cells leads to contact inhibition of locomotion via repolarization of forces. *Dev. Cell* 34, 421-434. 10.1016/j.devcel.2015.06.012 26235046 PMC4552721

[DMM024661C47] Shively J. E. and Conrad H. E. (1976). Formation of anhydrosugars in the chemical depolymerization of heparin. *Biochemistry* 15, 3932-3942. 10.1021/bi00663a005 9127

[DMM024661C48] Shworak N. W., Shirakawa M., Mulligan R. C. and Rosenberg R. D. (1994). Characterization of ryudocan glycosaminoglycan acceptor sites. *J. Biol. Chem.* 269, 21204-21214. 7520439

[DMM024661C49] Simões-Costa M. and Bronner M. E. (2015). Establishing neural crest identity: a gene regulatory recipe. *Development* 142, 242-257. 10.1242/dev.105445 25564621 PMC4302844

[DMM024661C50] Stachtea X. N., Tykesson E., van Kuppevelt T. H., Feinstein R., Malmström A., Reijmers R. M. and Maccarana M. (2015). Dermatan sulfate-free mice display embryological defects and are neonatal lethal despite normal lymphoid and non-lymphoid organogenesis. *PLoS ONE* 10, e0140279 10.1371/journal.pone.0140279 26488883 PMC4619018

[DMM024661C51] Subramanian A., Tamayo P., Mootha V. K., Mukherjee S., Ebert B. L., Gillette M. A., Paulovich A., Pomeroy S. L., Golub T. R., Lander E. S. et al. (2005). Gene set enrichment analysis: a knowledge-based approach for interpreting genome-wide expression profiles. *Proc. Natl. Acad. Sci. USA* 102, 15545-15550. 10.1073/pnas.0506580102 16199517 PMC1239896

[DMM024661C52] Syx D., Van Damme T., Symoens S., Maiburg M. C., van de Laar I., Morton J., Suri M., Del Campo M., Hausser I. and Hermanns-Lê T. (2015). Genetic heterogeneity and clinical variability in musculocontractural Ehlers–Danlos syndrome caused by impaired Dermatan Sulfate biosynthesis. *Hum. Mutat.* 36, 535-547. 10.1002/humu.22774 25703627

[DMM024661C53] Thelin M. A., Svensson K. J., Shi X., Bagher M., Axelsson J., Isinger-Ekstrand A., van Kuppevelt T. H., Johansson J., Nilbert M. and Zaia J. (2012). Dermatan sulfate is involved in the tumorigenic properties of esophagus squamous cell carcinoma. *Cancer Res.* 72, 1943-1952. 10.1158/0008-5472.CAN-11-1351 22350411 PMC3328612

[DMM024661C54] Thelin M. A., Bartolini B., Axelsson J., Gustafsson R., Tykesson E., Pera E., Oldberg A., Maccarana M. and Malmstrom A. (2013). Biological functions of iduronic acid in chondroitin/dermatan sulfate. *FEBS J.* 280, 2431-2446. 10.1111/febs.12214 23441919 PMC3717172

[DMM024661C55] Thiery J. P., Acloque H., Huang R. Y. J. and Nieto M. A. (2009). Epithelial-mesenchymal transitions in development and disease. *Cell* 139, 871-890. 10.1016/j.cell.2009.11.007 19945376

[DMM024661C56] Trainor P. A. and Andrews B. T. (2013). Facial dysostoses: etiology, pathogenesis and management. *Am. J. Med. Genet. C Semin. Med. Genet.* 163, 283-294. 10.1002/ajmg.c.31375 24123981 PMC3870197

[DMM024661C57] Trowbridge J. M. and Gallo R. L. (2002). Dermatan sulfate: new functions from an old glycosaminoglycan. *Glycobiology* 12, 117R-125R. 10.1093/glycob/cwf066 12213784

[DMM024661C58] Tsai J. H. and Yang J. (2013). Epithelial-mesenchymal plasticity in carcinoma metastasis. *Genes Dev.* 27, 2192-2206. 10.1101/gad.225334.113 24142872 PMC3814640

[DMM024661C59] Tsuji T., Ibaragi S. and Hu G.-F. (2009). Epithelial-mesenchymal transition and cell cooperativity in metastasis. *Cancer Res.* 69, 7135-7139. 10.1158/0008-5472.CAN-09-1618 19738043 PMC2760965

[DMM024661C60] Tucker R. (1986). The role of glycosaminoglycans in anuran pigment cell migration. *J. Embryol. Exp. Morphol.* 92, 145-164. 3723060

[DMM024661C61] Tucker A. S. and Slack J. M. W. (2004). Independent induction and formation of the dorsal and ventral fins in Xenopus laevis. *Dev. Dyn.* 230, 461-467. 10.1002/dvdy.20071 15188431

[DMM024661C62] Veiga S. S., Elias M. C. Q. B., Gremski W., Porcionatto M. A., da Silva R., Nader H. B. and Brentani R. R. (1997). Post-translational modifications of alpha5beta1 integrin by glycosaminoglycan chains. The alpha5beta1 integrin is a facultative proteoglycan. *J. Biol. Chem.* 272, 12529-12535. 10.1074/jbc.272.19.12529 9139704

[DMM024661C64] Wang C., Gong B., Bushel P. R., Thierry-Mieg J., Thierry-Mieg D., Xu J., Fang H., Hong H., Shen J. and Su Z. (2014). The concordance between RNA-seq and microarray data depends on chemical treatment and transcript abundance. *Nat. Biotechnol.* 32, 926-932. 10.1038/nbt.3001 25150839 PMC4243706

[DMM024661C65] Woods A., Longley R. L., Tumova S. and Couchman J. R. (2000). Syndecan-4 binding to the high affinity heparin-binding domain of fibronectin drives focal adhesion formation in fibroblasts. *Arch. Biochem. Biophys.* 374, 66-72. 10.1006/abbi.1999.1607 10640397

[DMM024661C66] Zhang L., Müller T., Baenziger J. U. and Janecke A. R. (2010). Congenital disorders of glycosylation with emphasis on loss of dermatan-4-sulfotransferase. *Prog. Mol. Biol. Transl. Sci.* 93, 289-307. 10.1016/S1877-1173(10)93012-3 20807649

[DMM024661C67] Zhang D., Ighaniyan S., Stathopoulos L., Rollo B., Landman K., Hutson J. and Newgreen D. (2014). The neural crest: a versatile organ system. *Birth Defects Res. C. Embryo Today* 102, 275-298. 10.1002/bdrc.21081 25227568

